# Computational Model Study of the Experimentally Suggested Mechanism for Nitrogenase

**DOI:** 10.1021/acs.jpcb.3c07675

**Published:** 2024-01-18

**Authors:** Per E. M. Siegbahn

**Affiliations:** Department of Organic Chemistry, Arrhenius Laboratory, Stockholm University, SE-106 91 Stockholm, Sweden

## Abstract

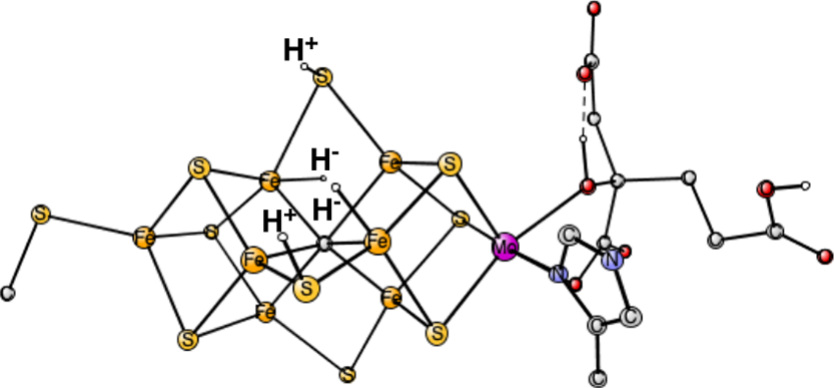

The mechanism for N_2_ activation in the E_4_ state of nitrogenase was investigated by model calculations. In the experimentally suggested mechanism, the E_4_ state is obtained after four reductions to the ground state. In a recent theoretical study, results for a different mechanism have been found in excellent agreement with available Electron Paramagnetic Resonance (EPR) experiments for E_4_. The two hydrides in E_4_ leave as H_2_ concertedly with the binding of N_2_. The mechanism suggested differs from the experimentally suggested one by a requirement for four activation steps prior to catalysis. In the present study, the experimentally suggested mechanism is studied using the same methods as those used in the previous study on the theoretical mechanism. The computed results make it very unlikely that a structure obtained after four reductions from the ground state has two hydrides, and the experimentally suggested mechanism does therefore not agree with the EPR experiments for E_4_. Another structure with only one hydride is here suggested to be the one that has been observed to bind N_2_ after only four reductions of the ground state.

## 1 Introduction

**Figure 1:**
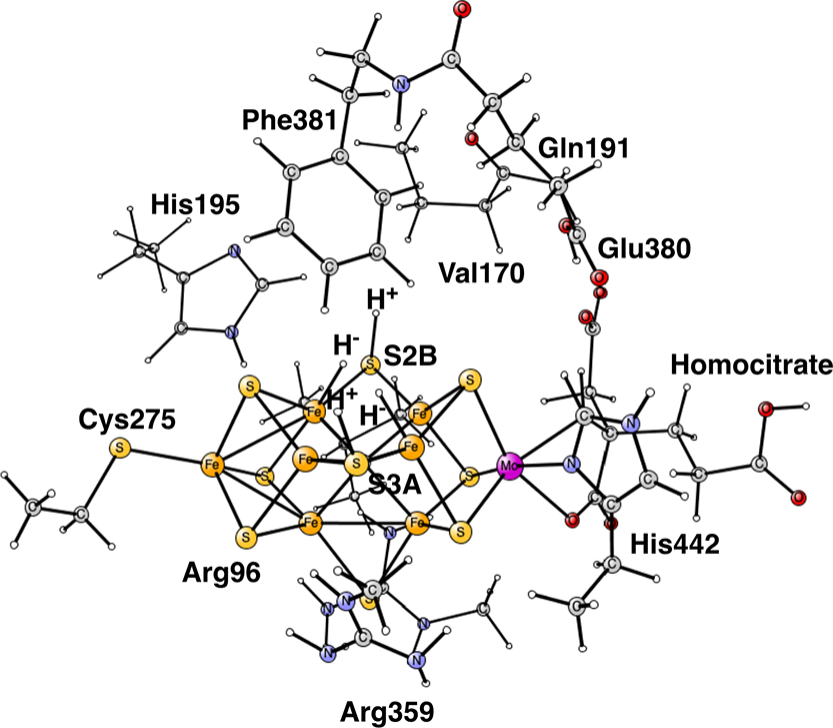
Model used for the present study shows which amino acids were included. The model is built on the X-ray structure 3U7QS^2^ and is shown here after four reductions from the ground state with two hydrides and two protonated sulfides.

Nitrogenases are the only enzymes in nature that can convert nitrogen in air into useful products. To accomplish this task, the most common form uses a complicated cofactor consisting of seven irons and one molybdenum connected by sulfides.^1^ An unusual feature of the cofactor is a centrally bound carbide.^2^ Another one is a homocitrate ligand bound to the molybdenum. An optimized structure obtained after four reductions (E_4_) is shown in Figure 1.

A plausible mechanism for N_2_ activation has been very difficult to obtain, but it has long been known that the first step in N_2_ activation occurs after four reduction steps in the catalytic cycle in a state termed E_4_.^3^ A breakthrough in the understanding of activation occurred using EPR a decade ago. The main features obtained in the experimental EPR study of the N_2_ activation in E_4_ are the following^4−6^

1. E_4_ should have two hydrides.

2. These hydrides should form H_2_ in a concerted process with N_2_ binding.

3. The process is reversible by changing the pressures of H_2_ and N_2_.

4. The barriers for formation of H_2_ from a hydride and a proton must be higher than the one for the allowed formation of H_2_ from the two hydrides.

5. A mutation of the second shell Val70 significantly disturbs the nitrogenase activity.^7^

The EPR experiment explained the surprising fact that for each N_2_, there is a release of one H_2_. The overall reaction is
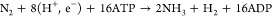


In the experimentally suggested mechanism, E_4_ is obtained after four reductions of the ground state E_0_.^4−6^ Since two hydrides are formed, the redox state of E_4_ should be the same as the one of E_0_, which is known to be (Mo^3+^5Fe^3+^2Fe^2+^).^4−6^ However, theoretical modeling studies have suggested that four preactivation steps are needed before catalysis starts.^8^ That suggestion implies that E_4_ is obtained, not by four, but by eight reductions of the ground state. With eight reductions, the redox state of E_4_ instead is (Mo^3+^7Fe^2+^), which is the lowest possible stable redox state for the cofactor. With the eight reductions, four more positions to place the protons, always accompanied by the reductions, are needed than those for the experimental mechanism. For that reason, it was initially suggested that the carbide should be protonated three times.^9^ However, it was later shown by a modeling study that with a release of a sulfide from the cofactor, the carbide would only be protonated once.^10^ Recently, it was found experimentally that the carbide is not protonated at all.^11^

A mechanism for nitrogen activation in E_4_ without carbide protonation has recently been suggested by model calculations.^12^ That mechanism agrees with all of the above experimental findings for the E_4_ state. Point 4 above is particularly significant since it means that several independent requirements on the mechanism are fulfilled.

The experimentally suggested mechanism for nitrogen activation in the E_4_ state is quite different from the theoretically suggested one since it is built on the premise that E_4_ in the catalytic cycle is reached after only four reductions of the ground state,^4−6^ instead of the eight reductions required in the theoretical mechanism. In the present modeling study, the experimental mechanism will be investigated by the same methods as were used recently for the theoretical mechanism.^12^ The purpose of the study is to investigate if the experimentally suggested structure fulfills the requirements set by the EPR experiments for E_4_.

There are many theoretical studies of the E_4_ state of nitrogenase. The most abundant ones are the ones by Cao and Ryde,^13^ Pang and Björnsson,^14^ and Dance.^15^ However, none of them discuss the energetics of H_2_ release in E_4_, which is the subject of this paper, and they will therefore not be discussed further here.

## 2 Methods

The methods used in the present study are exactly the same as those used in the recent study of the theoretical mechanism of the E_4_ state.^12^ They have been tested on a large number of redox enzymes over the past decades.^16−18^ In particular, the mechanism of seven redox enzymes has been compared to available experiments with errors of usually less than 3 kcal/mol.^18^ Those enzymes were photosystem II, cytochrome c oxidase, NiFe and FeFe hydrogenases, NiFe-CO dehydrogenase, multicopper oxidase, and acetyl-CoA synthase. The electronic structure method is built on the DFT functional B3LYP^19^ but with the fraction of exact exchange changed from 20 to 15%. Geometries were optimized with the rather small lacvp* basis set, and the final energies were obtained with a large cc-pvtz(-f) basis. For zero-point effects and determination of transition states, Hessians were computed by using B3LYP with lacvp*. Dispersion effects were obtained with the empirical D2 correction.^20^ Cluster modeling of the enzyme active site was used,^21^ with a model of about 170 atoms, essentially the same as in the previous study.^12^ Solvation effects were included with a dielectric constant of 4.0.^22^ The spin-coupling used for all states studied here was (−2,–3,–4), where the nomenclature shows the irons that have negative spins. The experimental numbering of the irons is used. The spin state is a doublet. The Jaguar and Gaussian programs were used.^22,23^ Since the BP86 functional^24^ was used in the theoretical part of the experimental paper on the mechanism,^25^ it was also used here for comparisons.

## 3 Results

**Figure 2:**
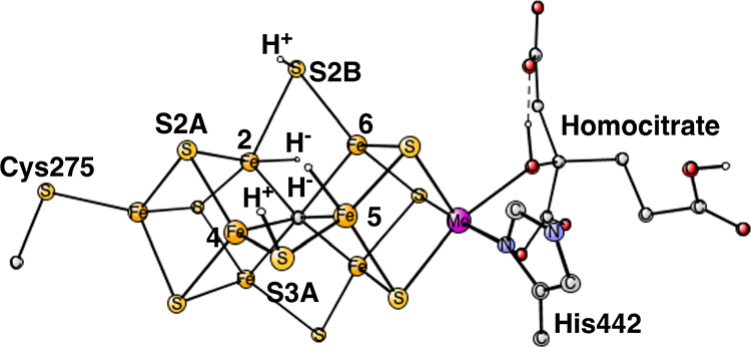
B3LYP optimized structure obtained after four reductions from the ground state. It follows as closely as possible the experimentally suggested structure,^25^ as described in the text. It has two hydrides and two protonated sulfides. The numbering of the irons follows that of the X-ray.

The present study is an investigation of the experimentally suggested mechanism for nitrogenase.^4−6^ Following that suggestion, the activation of N_2_ takes place after four reductions of the ground state E_0_, in a state commonly referred to as E_4_. In the EPR study, it was found that there are two hydrides in E_4_. With four reductions, it means that two groups are protonated in E_4_, which are suggested to be the belt sulfides S2B and S3A, see Figures 1 and 2.^25^ The two hydrides were found to disappear from the cluster as N_2_ was activated, suggesting a concerted process. The structure in Figure 1 was obtained using BP86, while that in Figure 2 was found using B3LYP following the experimental suggestion for the structure. The structures are quite similar and the spin states are doublets. It is important to note that the B3LYP structure in the figure is much higher in energy by +14.7 kcal/mol than the one with only one hydride and three protonated sulfides using that functional.^17,26^

The first part of the present study reports the results using the B3LYP functional with 15% exact exchange. For details of the methods, see Section 2. The first step in the N_2_ activation should be the formation of H_2_ from the two hydrides. To obtain the reaction energy, the two hydrides were removed from the cluster and placed at a long distance away as H_2_. The reaction was found to be exergonic by −47.9 kcal/mol, including a gain of −8.4 kcal/mol for the translational entropy of free H_2_. The result is in line with previous studies using this functional.^17,26^ In the EPR study, the process should be reversible by varying the pressures of H_2_ and N_2_. For the binding of N_2_, see further below.

**Figure 3:**
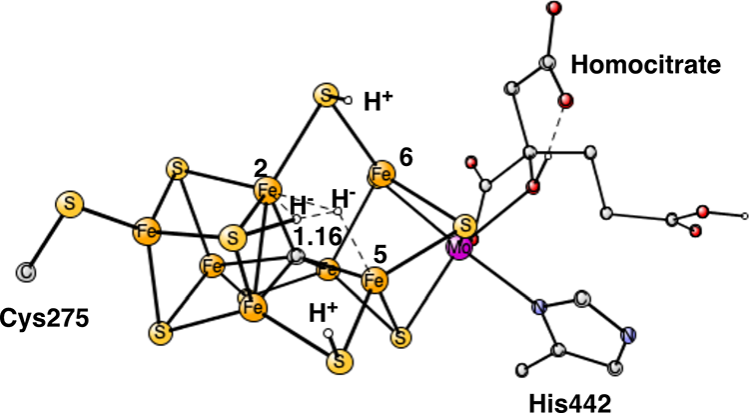
Optimized TS for H_2_ formation from the two hydrides.

The release pathway for H_2_ was studied by varying the H–H distance in steps of 0.1–0.2 Å. As the distance was decreased between the hydrogens, the hydride originally bound between Fe2 and Fe6 moved toward S2A and the energy goes down by −6.4 kcal/mol. That result agrees with the previous ones showing that a structure with three protonated sulfides and one hydride is lower in energy than one with two hydrides.^17^ It should be added that the protonation of S2A does not give the lowest energy structure. As the H–H distance was shortened further, a TS for H_2_ formation was reached. A full TS optimization was performed, and the H–H distance was found to be 1.16 Å; see Figure 3. The imaginary frequency is 1177 cm^–1^. There is a small barrier of 4.4 kcal/mol counted from the starting point with two hydrides. However, from the lowest point on the pathway, with a protonated S2A, the barrier is 10.8 kcal/mol. When the H–H distance is shortened further to 1.10 Å, the energy goes down by −1.1 kcal/mol. For H–H = 1.00 Å, H_2_ automatically moves away from the cluster in the optimization without any additional barrier. At the end of the optimization with fixed H–H = 1.00 Å, the distance to the nearest sulfide (S2A) is 2.6 Å and to the nearest iron (Fe1) 4.1 Å, and the energy is −27.5 kcal/mol lower than the TS energy. That behavior is totally different from what was suggested based on the EPR results.^4−6^

To complete the picture, the binding of N_2_ was investigated for the structure where H_2_ has been released. Binding to Fe6 has been favored experimentally, but no binding was found when the translational entropy loss of +9.9 kcal/mol was included. A similar result was obtained for the binding on Fe4.

As mentioned above, the B3LYP functional with 15% exact exchange has been tested for the mechanisms of seven enzyme redox reactions, actually all the ones studied so far.^18^ The results show errors of usually 3 kcal/mol or less compared with available experiments. In contrast, the BP86 functional^24^ used in the combined experimental and theoretical study of the nitrogenase mechanism^25^ has not been tested on any enzyme redox mechanism. However, for completeness, the nitrogenase mechanism in E_4_ has here been studied by also using BP86.

The E_4_ structure obtained using the BP86 functional is shown in Figure 1. It is very similar to the B3LYP structure. It can be noted that the hydride bound between Fe2 and Fe6 points in a different direction for the lowest energy structure than for the one in the previous study.^25^ The energy difference is small (−3.5 kcal/mol) and is not significant for the present study.

When the two hydrides are removed from the BP86 structure, forming a free H_2_, the energy goes down by −32.5 kcal/mol, including the translational entropy gain of −8.4 kcal/mol. This exergonicity is smaller than for B3LYP with −47.9 kcal/mol, see above but still very different from what was suggested by the EPR experiments, which indicate a reversible release of H_2_ and binding of N_2_. In the previous study,^25^ no energies for this process were given, but only a sketch of the results was shown in a figure. From that figure, it is clear that very different results were obtained there than the ones obtained here, even though the BP86 functional was used in both studies and the E_4_ structures are very similar.

For locating an approximate TS for H_2_ formation, the H–H bond was varied in steps, just as for B3LYP, described above. A linear scan was done and the highest point was found for a distance of 1.10 Å. The approximate barrier is +9.1 kcal/mol compared to +10.8 kcal/mol for B3LYP.

An important feature of the suggested mechanism in the previous study^25^ was that a big barrier was found for the loss of H_2_. As described above, with the B3LYP functional, there is no barrier for the release after the TS has been passed. The question is whether there is a barrier using BP86. A very large barrier of almost 20 kcal/mol is required in the suggested mechanism, in order to prevent the release for a sufficiently long time to allow a quite strong bond formation of N_2_. To investigate this question, the H–H bond is shortened from 1.10 Å in the TS to 0.90 Å and then to 0.70 Å. Just as for the B3LYP functional, no additional barrier was found. At the end point, the nearest distance to sulfur is 4.0 Å, and to the nearest iron is 4.1 Å. A difference between the results for BP86 and B3LYP is that with BP86 the protonated S2A is not lower in energy than the dihydride starting point.

In the suggested mechanism,^25^ a strong binding of N_2_ is required to the cluster, where H_2_ has been removed. In the present study, no binding at all is found, using either B3LYP, see above, or BP86. Binding on Fe6 and Fe4 was investigated also for BP86. The lack of binding of N_2_ to the type of experimentally suggested structure in Figures 1 and 2, is in line with another recent theoretical study.^27^ In that study, it was concluded that for the type of structure suggested experimentally, no functional gives favorable binding to any E_*n*_ (*n* = 0,4) state. Several DFT functionals were used.

**Figure 4:**
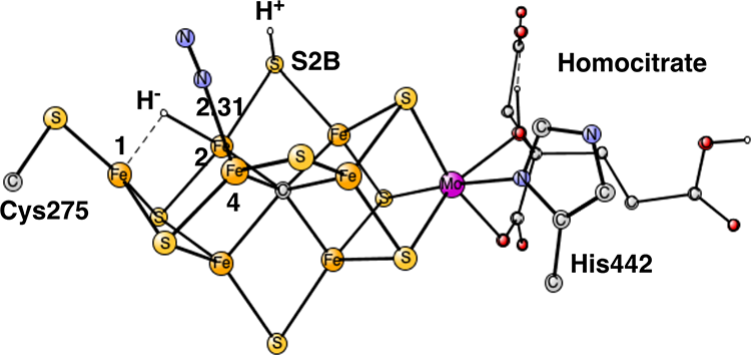
Optimized A_4_ structure with bound N_2_. There is only one hydride in this structure.

The best structure obtained after only four reductions in the previous study has only one hydride.^10^ The state was termed A_4_. An important feature of that A_4_ structure is that it is preceded (in A_4_) by a loss of a sulfide, leading to a large structural change. It was investigated here whether N_2_ could bind to that structure. Indeed, a weak binding to Fe4 was found, see Figure 4. It is the same position that was found to bind and activate N_2_ four reductions later in E_4_.^12^ The enthalpic binding energy is calculated to be −9.8 kcal/mol. In the case of a strong binding, the entire translational entropy of +9.9 kcal/mol is assumed to be lost upon the binding. In the present case of a very weak binding, it is reasonable to assume that some of that entropy is kept. A free energy of binding of about 5 kcal/mol appears reasonable. It is here speculated that it is the structure in Figure 4 that has been considered as an activated N_2_ structure in the investigation by Lowe and Thorneley.^3^

## 4 Conclusions

The key step in the nitrogenase mechanism is the nitrogen activation in the E_4_ state of the catalytic cycle. EPR experiments have outlined the main features of the activation, see Section 1.^4−6^ In combination with other experimental results,^3^ a structure for E_4_ with two hydrides and two protonated sulfides was suggested. In a combined theoretical and experimental study, the details of the mechanism were studied.^25^ In another theoretical paper,^12^ a different mechanism has recently been suggested, which agrees with all experimental findings for E_4_. In that mechanism, four preactivation steps were suggested. No such preactivation is included in the experimentally suggested mechanism, where N_2_ should be activated after only four reductions of the ground state.

In the present study, an investigation has been performed for the experimentally suggested mechanism using the same methods as used for the theoretical mechanism based on B3LYP with 15% exact exchange.^12^ In contrast, the BP86 functional was used in the previous combined theoretical and experimental study.^25^ Therefore, also the results for the BP86 functional were obtained in the present study.

The present study shows that the experimentally suggested structure for E_4_ with two hydrides, obtained after four reductions of the ground state (A_4_), leads to a completely different behavior than what has been observed by EPR for E_4_ in the catalytic cycle.^4−6^ Experimentally, the release of H_2_ and binding of N_2_ occurs in a concerted step in E_4_, which can be reversed by changing the pressures of H_2_ and N_2_. In contrast, the calculations on the A_4_ state with two hydrides give a very exergonic release of H_2_ by −47.9 kcal/mol using B3LYP (15%), and N_2_ does not bind at all in the product. Possible errors in the calculations of this order of magnitude can safely be ruled out.^18^ The conclusion drawn here is that the A_4_ state obtained after four reductions of the ground state is not the same structure as the one observed by EPR for E_4_ in the catalytic cycle. Instead, it is here suggested that the structure that binds N_2_ after only four reductions^3^ is the one in Figure 4 with only one hydride.

## Supporting Information Available

Coordinates for all structures (PDF)
