# Supplementary material for: Computational Model Study of the Experimentally Suggested Mechanism for Nitrogenase
Source: J Phys Chem B. 2024 Jan 18;128(4):985–9. doi: 10.1021/acs.jpcb.3c07675 (PMC10839828; doi:10.1021/acs.jpcb.3c07675)
Supplement: Supplementary file 1 — jp3c07675_si_001.pdf [file jp3c07675_si_001.pdf]

Supporting Information:

Computational Model Study of the Experimentally Suggested Mechanism for Nitrogenase

Per E. M. Siegbahn*

Department of Organic Chemistry, Arrhenius Laboratory, Stockholm University,

SE-106 91, Stockholm, Sweden. Email:per.siegbahn@su.se

The B3LYP optimized E_4 structure in **Figure 2.** # means fixed atom
Energies: E= -8163.461838, solv = -0.200141, disp = -227.51 Z_0 = 835.85

Mo1	16.6983450875	-5.7232093996	53.2699724176
Fe2	9.4480958422	-7.0405263782	55.8929295389
Fe3	11.8328440227	-5.2725361606	55.7179745397
Fe4	11.0695770443	-6.4780675864	53.2655625684
Fe5	12.2118343397	-7.9312316104	55.3129522444
Fe6	14.8201073592	-7.3072040015	54.8248466213
Fe7	14.4574933004	-4.7956977816	55.3343239894
Fe8	13.7549898727	-5.9882345966	52.7427270479
C9	17.9289620026	-2.1341463015	53.9958907674
H10	18.1022902995	-1.1202781216	53.6283175353
H11	16.8460522850	-2.2492646474	54.1193005517
C12	18.4356127588	-3.1887739143	52.9796769099
C13	19.8665712128	-2.8449095530	52.5201908171
H14	20.5191837676	-2.8868695144	53.3963511791
H15	19.8643285854	-1.8178943548	52.1411101641
C16	20.3823231898	-3.7965311317	51.4181139494
H17	20.0378485412	-3.4861758194	50.4304064589
H18	19.9917609965	-4.8016114330	51.6189203134
C19	17.5995996083	-3.4050176022	51.6778453007
O20	17.2420266618	-4.6493749175	51.4784209826
O21	17.4437118006	-2.4783665729	50.8875234168
O22	18.4430133030	-4.4563899630	53.6595358942
C23	13.0129816052	-6.3286068387	54.5245430245
S24	11.3984575522	-7.0242021607	57.2759664228
S25	16.5380474084	-6.0753187005	55.6806424716
S26	9.7702907065	-4.9247429471	54.6021767094
S27	13.3501148874	-4.0124471578	57.2757560883
S28	14.0781801933	-9.6067739661	55.2593255262
S29	14.9251391076	-3.9379997017	53.2126239978
S30	10.2939379760	-8.6276701691	54.1005693709
S31	15.3736058038	-7.6122315103	52.5327560010
S32	12.0298312395	-6.0844577951	51.1708188504

H33	13.6351749395#	-2.1902102008#	49.0425570129#
C34	13.2979258921#	-2.0241168994#	50.0550232009#
H35	13.9892287977	-2.5660873876	50.7065834399
H36	13.3427203770	-0.9534466207	50.2873154379
N37	11.9381872214	-2.5445321201	50.1978990859
H38	11.3366676630	-2.5363604973	49.3878993999
C39	11.3732324876	-2.9682494570	51.3383914962
N40	11.9907792134	-2.8667990544	52.5023818028
H41	11.6299682059	-3.4097176170	53.2902889256
H42	12.9982195929	-2.7232580354	52.5486254548
N43	10.0729875857	-3.3950642690	51.2998518694
H44	9.9098913552	-4.0593863134	50.5485637276
H45	9.7707029919	-3.8113242923	52.1872436085
H46	7.9284045819	0.3671904605	59.4090278005
C47	8.2032240780	-1.3604200003	60.7499816455
H48	8.2719003923	-1.4824323515	61.8415558237
H49	7.2421260213	-1.8048838633	60.4571789590
C50	9.3003414972	-2.1549121352	60.1227906908
N51	10.6191668293	-1.9840587165	60.4987691788
C52	9.1926263066	-3.1907692771	59.2223118913
H53	8.3500363629	-3.6376438785	58.7156855779
C54	11.2899669403	-2.9021111182	59.8312342834
H55	12.3586141129	-3.0529566965	59.8645773615
N56	10.4736931487	-3.6629166697	59.0501376420
H57	19.3525874128	-4.4461928162	57.3752980926
H58	16.7359445274	-8.8350283333	50.8068936719
C59	18.4302733364	-7.4441286042	50.6040860072
H60	17.8384079300	-6.5268495660	50.5889273645
H61	19.2338491409	-7.3069421569	49.8666933688
C62	19.0601029294	-7.5969870363	51.9560225303
N63	18.4624525369	-7.1278490018	53.1166130751
C64	20.2569159896	-8.1783358551	52.2977172992
H65	21.0191838715	-8.6520839037	51.6985184254
C66	19.2691055553	-7.4101590065	54.1260961997
H67	19.1226189959	-7.1403901127	55.1618892292
N68	20.3654057306	-8.0587085511	53.6725989138
H69	21.1445285399	-8.3319589680	54.2524273159
H70	7.0067260946	-10.4998713276	56.9136973944
C71	6.1750531141#	-9.8249625954#	56.6900003513#
H72	5.5309556515#	-10.2322289947#	56.0424863628#
H73	5.6932327119#	-9.5371413783#	57.6127206446#
S74	7.5647595098	-7.4121412495	57.1339798938
H75	10.7633592043	-4.4034098082	58.4202766848
H76	18.6888550513	-4.2081355548	54.6653677669
C78	18.5400332537	-2.2506137329	55.4296544919
O79	18.9602316624	-3.4237291212	55.7952921627
O80	18.5446720676	-1.2359984478	56.1423326175
C81	21.8761200167	-3.9061503523	51.4065587071
O82	22.6416446645	-3.7340641337	50.4783427236
O83	22.3208177416#	-4.2658019929#	52.6398579840#

H84	15.7024659776#	-0.1996310399#	61.4912300214#
C85	14.8549930764#	-0.0829919162#	60.8320009473#
H86	13.9165020076#	-0.2867340019#	61.3260960181#
H87	14.8294323163	0.9924776156	60.5864313578
C88	15.0599869109#	-0.8751940406#	59.5441970301#
H89	14.3527623494	-0.5240371506	58.7828352266
H90	14.8210948043	-1.9305803587	59.7157743456
C91	16.4835972769	-0.7984101808	58.9836213694
H92	16.9001669508	0.2127150452	59.1088571540
H93	16.4911505433	-0.9833633925	57.9031369436
C94	17.4481547622	-1.7974103647	59.6347016584
O95	17.1822649080	-2.4113052810	60.6730814798
N96	18.6367952534	-1.9306988949	58.9830738065
H97	19.1861022681	-2.7363280289	59.2585269788
H98	18.7062300174	-1.6535485425	57.9951339703
C99	10.6519929840#	0.0100038759#	53.3460009415#
H100	11.1636989831#	0.9592720146#	53.2872700916#
H101	9.5863320119#	0.1850130761#	53.3579369535#
H102	10.9236805785	-0.5873543362	52.4755133443
C103	11.0476301039	-0.6530429587	54.6852510305
H104	10.7586925735	-1.7105044683	54.6553156762
C105	10.2772799396	-0.0128414604	55.8501435531
H106	9.1938012372	-0.1278515299	55.7200439598
H107	10.4958633851	1.0623715417	55.9248889392
H108	10.5480176616	-0.4773841476	56.8047003348
C109	12.5585448466	-0.5649151694	54.9429824225
H110	13.1465375169	-0.9715904503	54.1133192473
H111	12.8402305666	-1.1165082710	55.8465266984
H112	12.8631224971	0.4829323411	55.0788400217
H113	13.4550771320#	-12.2303889699#	53.1883131024#
C114	13.9110027930#	-11.6210550441#	52.4219888761#
H115	14.8321164161	-11.1682258942	52.7982806376
H116	14.0899772393	-12.1935393860	51.5081158201
N117	12.8427348199	-10.6355400332	52.2054920496
H118	12.2038973063	-10.4763545687	52.9847902976
C119	12.7044101051	-9.7871462380	51.1896901670
N120	13.7321981200	-9.5977067107	50.3163478886
H121	13.5624591280	-8.8335990400	49.6688719997
H122	14.6312187275	-9.4745918728	50.7804655240
N123	11.5297166223	-9.1776948131	50.9758297057
H124	10.8460763597	-9.2877067903	51.7315304454
H125	11.6180851073	-8.1658731117	50.7390692010
H126	19.1948820194	-8.2145288005	61.7139089766
C127	19.5978930765#	-7.2298798566#	61.9900510785#
H128	20.6416651929	-7.3682191820	62.2914378140
C129	18.8441914655	-6.6640630253	63.1945832335
O130	19.4065797497	-6.1221595912	64.1428126877
C131	19.4918727873#	-6.2717981189#	60.7990648858#
H132	19.8421762479	-5.2761848465	61.0941346674
H133	18.4394267619	-6.1476654819	60.5257982983

C134	20.2692490326	-6.7573655639	59.5581699993
H135	20.0735986407	-7.8213492682	59.3790682140
H136	21.3517280951	-6.6852287737	59.7415308159
C137	20.0188564064	-6.0797479453	58.2019328096
O138	20.2997211455	-6.6451077146	57.1567573101
O139	19.5199673853	-4.8443588781	58.2781640818
N140	17.4897038431	-6.7873066410	63.0816721274
H141	17.1295341012	-7.1600200096	62.2115955041
C142	16.5368840413#	-6.0058840570#	63.8530430015#
H143	15.8862465258	-6.6824350708	64.4252741281
H144	17.1127714689	-5.4130233838	64.5681918692
C145	15.6695378019	-5.1051879392	62.9389851745
H146	16.2736324707	-4.2785455613	62.5495409774
H147	14.8705219055	-4.6675643353	63.5520234571
C148	15.0907103399	-5.8979498675	61.7832687204
C149	14.0655479975	-6.8363044978	61.9853109658
H150	13.6087054067	-6.9273595627	62.9695452107
C151	15.6513161422	-5.7824132189	60.5007777139
H152	16.4078489082	-5.0217130100	60.3217440747
C153	13.6175541803	-7.6429082107	60.9377974560
H154	12.8041196529	-8.3448309677	61.1014553218
C155	15.2177413874	-6.6030794634	59.4530233612
H156	15.6599883662	-6.5014553643	58.4653927549
C157	14.2003277489	-7.5366604651	59.6712919470
H158	13.8335593558	-8.1436007948	58.8503511485
H159	9.0037569068#	0.7050731035#	60.7752690799#
C160	8.1269793912#	0.1560810601#	60.4649993033#
H161	7.2836720882#	0.4592425672#	61.0677683410#
H269	18.2592771995#	-9.4987538402#	50.1505389943#
C270	17.5839765258#	-8.6559201021#	50.1520234506#
H271	17.2773631530#	-8.4419889356#	49.1387979674#
H273	23.2918667480	-4.3193490515	52.5665855704
H274	13.6466352558	-5.0127325197	58.1347524299
C275	6.6547745128	-8.5386006257	55.9994783984
H276	5.7778732745	-8.0012802522	55.6135681981
H277	7.2874299577	-8.7874500022	55.1414319184
H278	14.0978739664	-9.6298901311	56.6093513885
H279	12.3370212220	-4.0646063734	54.8889413041
H280	14.4048770105	-7.2484134447	56.2875700979

The B3LYP optimized H-H TS structure
Energies: E= -8163.456337, solv = -0.197082, disp = -225.69 Z_0 = 833.09

Mo1	16.7007600000	-5.9167240000	53.4156110000
Fe2	9.3604220000	-7.1704950000	55.6838320000
Fe3	12.0763430000	-6.0928290000	55.9225570000
Fe4	11.1157990000	-6.5978530000	53.2021890000
Fe5	11.8481200000	-8.5767430000	54.9455990000
Fe6	14.5878980000	-7.8207020000	55.1048980000

Fe7	14.7226180000	-5.2766530000	55.4834530000
Fe8	13.7894670000	-6.3285540000	52.8021650000
C9	17.8106680000	-2.2232170000	54.2210670000
H10	17.9432030000	-1.2178850000	53.8165330000
H11	16.7347610000	-2.3710340000	54.3734800000
C12	18.3199110000	-3.2899060000	53.2254510000
C13	19.7552820000	-2.9585890000	52.7621380000
H14	20.4182170000	-3.0441620000	53.6280560000
H15	19.7715550000	-1.9190600000	52.4176990000
C16	20.2405290000	-3.8791630000	51.6218310000
H17	19.7979770000	-3.5883190000	50.6676090000
H18	19.9337100000	-4.9066350000	51.8473270000
C19	17.4744210000	-3.4875800000	51.9318190000
O20	17.2011560000	-4.7351810000	51.6571280000
O21	17.2294340000	-2.5197780000	51.2151060000
O22	18.3231950000	-4.5524590000	53.9193830000
C23	12.8919020000	-6.8896270000	54.3685620000
S24	11.1344320000	-8.1051620000	57.2543160000
S25	16.5690760000	-6.6917630000	55.8194080000
S26	10.1538830000	-5.0265250000	54.7767490000
S27	13.2825510000	-4.1912220000	57.0480600000
S28	13.7466890000	-10.1303240000	55.4364000000
S29	14.8487010000	-4.2637290000	53.4226370000
S30	9.8709000000	-8.6712830000	53.7260820000
S31	15.4481450000	-7.9148210000	52.6986400000
S32	12.1341130000	-6.2170970000	51.1258250000
H33	13.6351750000	-2.1902100000	49.0425570000
C34	13.2979260000	-2.0241170000	50.0550230000
H35	14.0256430000	-2.5175590000	50.7060190000
H36	13.2922910000	-0.9489570000	50.2701390000
N37	11.9676070000	-2.6073740000	50.2308930000
H38	11.3407840000	-2.5922210000	49.4398260000
C39	11.4303930000	-3.0715560000	51.3690690000
N40	12.0460400000	-2.9958250000	52.5372030000
H41	11.6799840000	-3.5758490000	53.2980310000
H42	13.0502630000	-2.8431090000	52.5964740000
N43	10.1361360000	-3.5285040000	51.3210430000
H44	10.0124160000	-4.2233600000	50.5882140000
H45	9.8366550000	-3.9364580000	52.2102240000
H46	7.9182810000	0.3968360000	59.4164300000
C47	8.2884940000	-1.3597190000	60.6638450000
H48	8.5039960000	-1.5512140000	61.7253040000
H49	7.3331490000	-1.8554290000	60.4460090000
C50	9.3560210000	-1.9610380000	59.8141740000
N51	10.6779700000	-1.5960960000	59.9696410000
C52	9.2270070000	-2.8722330000	58.7917910000
H53	8.3767260000	-3.3923700000	58.3758110000
C54	11.3349080000	-2.2828870000	59.0549780000
H55	12.4011610000	-2.2629530000	58.8881400000
N56	10.5014590000	-3.0677220000	58.3142360000

H57	19.3113090000	-4.3689050000	57.4798990000
H58	16.7367220000	-8.8355660000	50.8065890000
C59	18.4270550000	-7.4399710000	50.6034400000
H60	17.8186430000	-6.5334970000	50.6288870000
H61	19.2029600000	-7.2729860000	49.8427800000
C62	19.1131380000	-7.6021490000	51.9276470000
N63	18.5465810000	-7.1875100000	53.1244610000
C64	20.3490930000	-8.1351010000	52.2048020000
H65	21.1062140000	-8.5577240000	51.5620640000
C66	19.4082100000	-7.4549070000	54.0926300000
H67	19.2913290000	-7.2170960000	55.1405330000
N68	20.5122590000	-8.0409610000	53.5760720000
H69	21.3279010000	-8.2941990000	54.1128420000
H70	7.0290460000	-10.4744450000	56.9002390000
C71	6.1750530000	-9.8249620000	56.6900000000
H72	5.5309560000	-10.2322290000	56.0424860000
H73	5.6932330000	-9.5371410000	57.6127210000
S74	7.4652710000	-7.2816380000	56.9771280000
H75	10.7805050000	-3.6954640000	57.5686260000
H76	18.5505420000	-4.3003740000	54.9216930000
C77	18.4670080000	-2.2896400000	55.6305450000
O78	18.8442870000	-3.4650730000	56.0318570000
O79	18.5470850000	-1.2481510000	56.2971660000
C80	21.7320900000	-3.8788430000	51.4768650000
O81	22.3910520000	-3.5985820000	50.4952070000
O82	22.3208180000	-4.2658020000	52.6398580000
H83	15.7024660000	-0.1996310000	61.4912300000
C84	14.8549930000	-0.0829920000	60.8320010000
H85	13.9165020000	-0.2867340000	61.3260960000
H86	14.8305600000	0.9923830000	60.5863940000
C87	15.0599870000	-0.8751940000	59.5441970000
H88	14.3733200000	-0.5039660000	58.7726790000
H89	14.7995880000	-1.9266510000	59.7131900000
C90	16.4944450000	-0.8216770000	59.0137430000
H91	16.9144440000	0.1896820000	59.1219730000
H92	16.5308640000	-1.0349430000	57.9391170000
C93	17.4327730000	-1.8093910000	59.7192490000
O94	17.1111680000	-2.4313620000	60.7345960000
N95	18.6629130000	-1.9175270000	59.1407230000
H96	19.1951370000	-2.7299720000	59.4297380000
H97	18.7631100000	-1.6478130000	58.1541910000
C98	10.6519930000	0.0100040000	53.3460010000
H99	11.1636990000	0.9592720000	53.2872700000
H100	9.5863320000	0.1850130000	53.3579370000
H101	10.9232340000	-0.5917770000	52.4758600000
C102	11.0603520000	-0.6448280000	54.6846580000
H103	10.7554940000	-1.6989440000	54.6708840000
C104	10.3246040000	0.0307430000	55.8529880000
H105	9.2370340000	-0.0746430000	55.7521670000
H106	10.5558920000	1.1051860000	55.8922730000

H107	10.6142180000	-0.4086710000	56.8134000000
C108	12.5787860000	-0.5776790000	54.9076620000
H109	13.1409820000	-1.0050720000	54.0699700000
H110	12.8726230000	-1.1222000000	55.8117150000
H111	12.9047270000	0.4659850000	55.0222930000
H112	13.4550770000	-12.2303890000	53.1883130000
C113	13.9110030000	-11.6210550000	52.4219890000
H114	14.7351240000	-11.0392480000	52.8425460000
H115	14.2396350000	-12.2205890000	51.5680420000
N116	12.7624290000	-10.7768120000	52.0456930000
H117	12.0039310000	-10.7435820000	52.7211150000
C118	12.6831940000	-9.8627350000	51.0774420000
N119	13.7815050000	-9.5443520000	50.3481940000
H120	13.6394720000	-8.7201480000	49.7698250000
H121	14.6221270000	-9.4203390000	50.9185990000
N122	11.4978340000	-9.3074860000	50.7687710000
H123	10.7500950000	-9.4697370000	51.4470300000
H124	11.5721430000	-8.2869950000	50.5810000000
H125	19.2084860000	-8.2184880000	61.7072930000
C126	19.5978930000	-7.2298800000	61.9900510000
H127	20.6402090000	-7.3577550000	62.2999700000
C128	18.8280900000	-6.6845710000	63.1900990000
O129	19.3811360000	-6.1915540000	64.1700630000
C130	19.4918730000	-6.2717980000	60.7990650000
H131	19.8556310000	-5.2805680000	61.0925940000
H132	18.4394540000	-6.1321940000	60.5307590000
C133	20.2520360000	-6.7637730000	59.5549230000
H134	19.9983430000	-7.8093750000	59.3408590000
H135	21.3357320000	-6.7573610000	59.7435400000
C136	20.0367000000	-6.0195790000	58.2299870000
O137	20.3771220000	-6.5135410000	57.1669890000
O138	19.4870590000	-4.8141030000	58.3645800000
N139	17.4734940000	-6.7627840000	63.0429020000
H140	17.1110410000	-7.1123030000	62.1641880000
C141	16.5368840000	-6.0058840000	63.8530430000
H142	15.9158240000	-6.6912510000	64.4477960000
H143	17.1333990000	-5.4092910000	64.5486380000
C144	15.6240090000	-5.1074490000	62.9881150000
H145	16.2225540000	-4.3347050000	62.4926040000
H146	14.9190520000	-4.5983170000	63.6586710000
C147	14.8794930000	-5.9345480000	61.9622260000
C148	13.7996870000	-6.7454710000	62.3430690000
H149	13.4306460000	-6.6968610000	63.3665490000
C150	15.3294930000	-5.9956250000	60.6342820000
H151	16.1326480000	-5.3333540000	60.3171100000
C152	13.1951220000	-7.6066920000	61.4280680000
H153	12.3491580000	-8.2180260000	61.7325450000
C154	14.7369220000	-6.8715440000	59.7177540000
H155	15.0997720000	-6.9157140000	58.6945030000
C156	13.6705790000	-7.6819880000	60.1162770000

H157	13.1917790000	-8.3479360000	59.4061000000
H158	9.0037570000	0.7050730000	60.7752690000
C159	8.1269790000	0.1560810000	60.4649990000
H160	7.2836720000	0.4592430000	61.0677680000
H161	18.2592770000	-9.4987540000	50.1505390000
C162	17.5839760000	-8.6559200000	50.1520240000
H163	17.2773630000	-8.4419890000	49.1387980000
H164	23.2808150000	-4.2321190000	52.4727790000
H165	13.6597630000	-4.9002940000	58.1335450000
C166	6.5820800000	-8.5332280000	55.9533930000
H167	5.6665660000	-8.0553080000	55.5790190000
H168	7.1880810000	-8.7868450000	55.0776100000
H169	13.3506490000	-10.1097330000	56.7270150000
H170	12.5879870000	-7.5074080000	57.2356500000
H171	13.5617710000	-7.0348750000	56.8321890000

The B3LYP optimized structure with S2A-H+
Energies: E= -8163.475010, solv = -0.198764, disp = -226.81 Z_0 = 836.15

Mo1	16.7188160803	-5.7558972757	53.2730277767
Fe2	9.4549611392	-7.1239435242	55.8556119356
Fe3	11.9325068037	-5.5686105620	55.7051976467
Fe4	11.1315689144	-6.5996544308	53.2729258403
Fe5	12.2810645632	-8.3032865064	55.2503714294
Fe6	14.8286699821	-7.4369771505	54.8415856195
Fe7	14.5592812459	-4.9341757543	55.3051818925
Fe8	13.7578641677	-6.0772639891	52.7185617104
C9	17.8941891943	-2.1384070767	54.0102982754
H10	18.0577161654	-1.1250612331	53.6372845472
H11	16.8118955641	-2.2660253817	54.1286814092
C12	18.4157012194	-3.1927691571	53.0019049983
C13	19.8484616028	-2.8416855880	52.5522641977
H14	20.4973833449	-2.8928784603	53.4310480528
H15	19.8479732774	-1.8106244880	52.1842248830
C16	20.3707746293	-3.7804826153	51.4420613810
H17	20.0164921490	-3.4666602263	50.4589446721
H18	19.9954122963	-4.7919078348	51.6382463709
C19	17.5860558833	-3.4116497348	51.6960965800
O20	17.2465568288	-4.6571101303	51.4819970513
O21	17.4173652723	-2.4760778152	50.9177305324
O22	18.4284854994	-4.4583214880	53.6860448874
C23	13.0371656014	-6.5149299661	54.4810309027
S24	11.2633562490	-7.9050777729	57.4828296725
S25	16.5825356913	-6.2269504866	55.6889612318
S26	9.9046515213	-4.9640941188	54.6903158252
S27	13.3245876279	-4.1719450941	57.1750169230
S28	14.2347350272	-9.8341298245	55.3093049139
S29	14.9076196454	-3.9990482088	53.2233622684
S30	10.2334300361	-8.7129598097	54.0609758486

S31	15.4011610858	-7.6730423121	52.5333225324
S32	12.0197629192	-6.1457973277	51.1488433785
H33	13.6351749275#	-2.1902102485#	49.0425570168#
C34	13.2979258793#	-2.0241168588#	50.0550232255#
H35	14.0078098718	-2.5362310953	50.7108645268
H36	13.3092342743	-0.9501734117	50.2752370418
N37	11.9524615119	-2.5833574776	50.2144945397
H38	11.3497213598	-2.5938973491	49.4049828385
C39	11.3882452182	-3.0274096973	51.3483031235
N40	11.9814630844	-2.9287150179	52.5272186529
H41	11.5928317891	-3.4924723741	53.2884911567
H42	12.9898862963	-2.7924539562	52.5953530297
N43	10.0957133393	-3.4818572764	51.2858500425
H44	9.9738921343	-4.1628038413	50.5404014541
H45	9.7912958337	-3.9079273883	52.1660721877
H46	7.9229223956	0.3783027235	59.4120788373
C47	8.2487424864	-1.3573838692	60.7107791940
H48	8.4014616643	-1.5208846904	61.7873440047
H49	7.2917678083	-1.8328504507	60.4571009304
C50	9.3413301287	-2.0214790754	59.9496797576
N51	10.6669055674	-1.7806976311	60.2516736020
C52	9.2391248512	-2.8906951500	58.8897915210
H53	8.3958866783	-3.3225660512	58.3718370027
C54	11.3526856011	-2.4989119172	59.3843421716
H55	12.4282029125	-2.5536129029	59.3131430735
N56	10.5353161579	-3.1880425929	58.5394688306
H57	19.3109876498	-4.4534104839	57.3843298156
H58	16.7399494876	-8.8278991141	50.8122277931
C59	18.4381580069	-7.4396504556	50.5806739316
H60	17.8452441112	-6.5228879198	50.5671136603
H61	19.2287735914	-7.3093233021	49.8280597415
C62	19.0930313802	-7.5786780518	51.9209016769
N63	18.5017700353	-7.1265510451	53.0911323343
C64	20.3059838870	-8.1366456787	52.2433735828
H65	21.0687169974	-8.5934300707	51.6317746102
C66	19.3259997805	-7.4001923343	54.0891140021
H67	19.1855708854	-7.1421320105	55.1287058206
N68	20.4291664748	-8.0236512436	53.6176519552
H69	21.2198439521	-8.2879300478	54.1857200829
H70	7.0071352904	-10.4992858004	56.9135450649
C71	6.1750531947#	-9.8249625791#	56.6900003100#
H72	5.5309556313#	-10.2322289893#	56.0424863795#
H73	5.6932327251#	-9.5371413477#	57.6127206419#
S74	7.5305175895	-7.3721247966	57.1055514667
H75	10.8378323181	-3.7835486726	57.7789409445
H76	18.6557315226	-4.2071731525	54.6934567837
C78	18.5009719612	-2.2416319086	55.4455061798
O79	18.9095698824	-3.4132738686	55.8258529055
O80	18.5124868572	-1.2187194791	56.1476826401
C81	21.8660100359	-3.8642778502	51.4227750935

O82	22.6260870920	-3.6379859653	50.5017273855
O83	22.3208177125#	-4.2658019895#	52.6398579805#
H84	15.7024659693#	-0.1996310551#	61.4912300294#
C85	14.8549931119#	-0.0829918864#	60.8320009346#
H86	13.9165020076#	-0.2867340019#	61.3260960181#
H87	14.8287456342	0.9919678622	60.5850757644
C88	15.0599868725#	-0.8751940519#	59.5441970387#
H89	14.3486811484	-0.5283335355	58.7842740476
H90	14.8326725184	-1.9330674104	59.7196774956
C91	16.4837635898	-0.7856330452	58.9830448012
H92	16.8907648422	0.2285148435	59.1141358857
H93	16.4959295648	-0.9652848787	57.9018293606
C94	17.4539411820	-1.7792081776	59.6351251952
O95	17.1948470952	-2.3807283904	60.6830718583
N96	18.6353516991	-1.9208808287	58.9747400853
H97	19.1882283225	-2.7226906987	59.2542159211
H98	18.6985831589	-1.6482274837	57.9844625578
C99	10.6519930493#	0.0100037925#	53.3460008367#
H100	11.1636989302#	0.9592720479#	53.2872701678#
H101	9.5863320170#	0.1850131058#	53.3579369713#
H102	10.9238183196	-0.5904365954	52.4767129740
C103	11.0500915934	-0.6504610875	54.6843749948
H104	10.7500470349	-1.7054688110	54.6593010748
C105	10.2970852176	0.0063382557	55.8512143287
H106	9.2114677841	-0.0986572091	55.7322317559
H107	10.5272396017	1.0798006444	55.9134329274
H108	10.5715478683	-0.4510735573	56.8079732239
C109	12.5641691311	-0.5758046457	54.9252611809
H110	13.1383553243	-0.9935196798	54.0915978045
H111	12.8516097942	-1.1222538336	55.8304362438
H112	12.8810940675	0.4694403624	55.0502073733
H113	13.4550771342#	-12.2303889742#	53.1883131004#
C114	13.9110027902#	-11.6210550483#	52.4219888825#
H115	14.8141095557	-11.1419944094	52.8072101444
H116	14.1172104147	-12.1991830218	51.5170994360
N117	12.8192061556	-10.6643359398	52.1777008953
H118	12.1482715455	-10.5406263577	52.9351003539
C119	12.6771703916	-9.8115866808	51.1653375789
N120	13.7176760183	-9.5764234703	50.3214011138
H121	13.5405701504	-8.8029754253	49.6866093759
H122	14.6014227344	-9.4324482420	50.8109226210
N123	11.4867709392	-9.2377362416	50.9284041510
H124	10.7915063047	-9.3744790854	51.6677560794
H125	11.5563555786	-8.2216810339	50.7048041976
H126	19.1945162987	-8.2142360173	61.7133530963
C127	19.5978930852#	-7.2298798085#	61.9900510783#
H128	20.6415977066	-7.3694066976	62.2910863464
C129	18.8444932573	-6.6674533158	63.1962364934
O130	19.4054333211	-6.1396077526	64.1532374628
C131	19.4918727355#	-6.2717981654#	60.7990648480#

H132	19.8485157603	-5.2777808520	61.0922355077
H133	18.4393838363	-6.1425613808	60.5297852493
C134	20.2601278378	-6.7619470652	59.5550189939
H135	20.0554661829	-7.8242862772	59.3755082394
H136	21.3441100537	-6.6988766642	59.7320135646
C137	20.0067967631	-6.0800012520	58.2017037596
O138	20.3059912050	-6.6320405095	57.1550696202
O139	19.4802654593	-4.8561202263	58.2843801667
N140	17.4897303130	-6.7790094656	63.0748891565
H141	17.1319368753	-7.1442316145	62.2009697263
C142	16.5368840471#	-6.0058840698#	63.8530430305#
H143	15.8823017687	-6.6885567332	64.4134999684
H144	17.1112867080	-5.4244239465	64.5786811073
C145	15.6726044073	-5.0893940368	62.9499987086
H146	16.2745423989	-4.2485520725	62.5888617797
H147	14.8616279424	-4.6744431682	63.5630934454
C148	15.1185267660	-5.8586075360	61.7668395992
C149	14.0991722211	-6.8114564955	61.9273877530
H150	13.6199540780	-6.9256995241	62.8984529260
C151	15.7081823060	-5.7133825135	60.5006102744
H152	16.4595613049	-4.9407795417	60.3536181237
C153	13.6907348113	-7.6096497350	60.8570327996
H154	12.8856922790	-8.3277703587	60.9878467343
C155	15.3185525125	-6.5284816337	59.4313451246
H156	15.7934422657	-6.4143607996	58.4601260151
C157	14.3107855422	-7.4816468916	59.6099107597
H158	13.9916990456	-8.1006938486	58.7776558840
H159	9.0037568920#	0.7050731175#	60.7752690968#
C160	8.1269794205#	0.1560810414#	60.4649992577#
H161	7.2836720955#	0.4592425552#	61.0677683573#
H269	18.2592772140#	-9.4987538285#	50.1505389948#
C270	17.5839764740#	-8.6559201227#	50.1520234816#
H271	17.2773631805#	-8.4419889215#	49.1387979621#
H273	23.2927073287	-4.2931203679	52.5632057309
H274	13.7390582091	-5.0675207831	58.0991637499
C275	6.6508942233	-8.5418981031	55.9878213695
H276	5.7740960236	-8.0195421314	55.5824392241
H277	7.2931320210	-8.7979857325	55.1386092998
H278	14.2697201029	-9.8085770781	56.6585080481
H279	11.8729260140	-6.7692073555	57.9054672232
H280	14.3064427657	-7.3573426659	56.2820556542

The B3LYP optimized structure for $E_4 – H_2$
Energies: E= -8162.353791, solv = -0.201746, disp = -220.93 Z_0 = 826.67

Mo1	16.4856966784	-5.7853710187	53.3297757671
Fe2	9.2123632891	-7.0197357103	55.8125873081
Fe3	11.6471734959	-5.4916351501	55.8615762791
Fe4	10.9302885639	-6.4384052477	53.2668349132

Fe5	11.8607295666	-8.0220904420	55.2675019343
Fe6	14.5291096049	-7.5613212952	54.5805920598
Fe7	14.2989408069	-5.0103691165	55.3852293691
Fe8	13.5674146968	-5.9730033101	52.7125333633
C9	17.8417816703	-2.1766674902	54.1205197895
H10	18.0528277606	-1.1827425313	53.7205010648
H11	16.7552555295	-2.2484571010	54.2503016496
C12	18.2988524083	-3.2794536806	53.1357404821
C13	19.7606303084	-3.0428124081	52.7003520541
H14	20.3976829334	-3.1326712528	53.5845952670
H15	19.8381655190	-2.0167935766	52.3244399012
C16	20.2276423484	-4.0226075099	51.6052004386
H17	19.7768935152	-3.7831481464	50.6405814698
H18	19.9190462147	-5.0361687911	51.8873976628
C19	17.4720550160	-3.4424220139	51.8253157527
O20	17.0849034352	-4.6709655031	51.5916226483
O21	17.3460446512	-2.4922831133	51.0575105490
O22	18.2033424138	-4.5344486437	53.8350538578
C23	12.8317342688	-6.4479112888	54.5290897229
S24	11.0956124725	-7.2746891363	57.3317121191
S25	16.2225066299	-6.4208736251	55.7189530586
S26	9.7405266253	-4.8113792692	54.6050511795
S27	13.0709203568	-3.8867300620	57.1003091476
S28	13.7006048788	-9.7211451609	55.3908060200
S29	14.7498658722	-3.9639535465	53.3519503050
S30	9.9610139188	-8.5611926654	53.9799542583
S31	15.2110976645	-7.5864249604	52.3062263548
S32	11.8293729039	-6.0859213518	51.1403739982
H33	13.6351750758#	-2.1902102883#	49.0425570727#
C34	13.2979256693#	-2.0241168071#	50.0550231294#
H35	13.9683703019	-2.5867461643	50.7107474041
H36	13.3635182949	-0.9569697575	50.2968503601
N37	11.9216458944	-2.5138890859	50.1759316983
H38	11.3375788345	-2.4837258469	49.3533781040
C39	11.3137731595	-2.9443334017	51.2911431783
N40	11.8885037460	-2.8844582894	52.4797943298
H41	11.4626017475	-3.4308214633	53.2332253608
H42	12.8978745959	-2.7698017039	52.5717638179
N43	10.0045985310	-3.3432753235	51.1966922014
H44	9.8720164304	-4.0176682301	50.4470292339
H45	9.6641200862	-3.7572327829	52.0687153436
H46	7.9082455908	0.3823880332	59.4167789853
C47	8.2803069894	-1.3607030868	60.7317373840
H48	8.9111892255	-1.5172480563	61.6143329210
H49	7.2909292804	-1.7549559138	61.0132485122
C50	8.8233236532	-2.2238879795	59.6325545719
N51	9.5697518776	-3.3417999857	59.9495373747
C52	8.5739596458	-2.1958060463	58.2776815694
H53	8.0241637818	-1.5136123834	57.6472248434
C54	9.7607530531	-3.9756613026	58.8087759646

H55	10.3025422560	-4.9010504725	58.6752146724
N56	9.1787853524	-3.3247138736	57.7663783449
H57	19.2808623027	-4.4039461071	57.4364366596
H58	16.7244230635	-8.8623792619	50.7840770786
C59	18.3806973934	-7.4333355413	50.6525687139
H60	17.7622607802	-6.5335041302	50.6256874705
H61	19.2145712445	-7.2622962896	49.9573927380
C62	18.9462265714	-7.5912698460	52.0322647101
N63	18.2720343160	-7.1802140356	53.1741179930
C64	20.1516177440	-8.1267768096	52.4171288962
H65	20.9615475430	-8.5495761538	51.8428653837
C66	19.0443328988	-7.4559314245	54.2128638329
H67	18.8378272714	-7.2180508456	55.2451950687
N68	20.1892104042	-8.0411822047	53.7982433587
H69	20.9560792927	-8.2783016015	54.4098947758
H70	7.0414609025	-10.4590036405	56.8934692711
C71	6.1750534788#	-9.8249627283#	56.6900005445#
H72	5.5309556048#	-10.2322287787#	56.0424862734#
H73	5.6932326829#	-9.5371413290#	57.6127206140#
S74	7.2801635998	-7.2104324426	57.0121095306
H75	9.2116834591	-3.6247717613	56.7948793359
H76	18.4452331372	-4.2923065359	54.8361627164
C78	18.4620757885	-2.2838946125	55.5463996798
O79	18.7890207239	-3.4750633270	55.9460428850
O80	18.5629296835	-1.2495550783	56.2230927497
C81	21.7177491021	-4.0427190328	51.4422211472
O82	22.3643324954	-3.9066112257	50.4221440996
O83	22.3208176654#	-4.2658019814#	52.6398579810#
H84	15.7024659581#	-0.1996310753#	61.4912300401#
C85	14.8549931568#	-0.0829918520#	60.8320009208#
H86	13.9165020076#	-0.2867340019#	61.3260960181#
H87	14.8276713259	0.9925609989	60.5856473904
C88	15.0599868215#	-0.8751940611#	59.5441970400#
H89	14.3567565561	-0.5242975494	58.7788625998
H90	14.8239106658	-1.9314621152	59.7140558951
C91	16.4884464518	-0.7833727526	58.9961096307
H92	16.8749566482	0.2434101820	59.0871461594
H93	16.5146424178	-1.0081991052	57.9237606456
C94	17.4717914260	-1.7280976365	59.6997392140
O95	17.2147125860	-2.2925949958	60.7678752456
N96	18.6652668429	-1.8709510326	59.0596929080
H97	19.2272274950	-2.6533893699	59.3734784348
H98	18.7347999288	-1.6328172240	58.0613901027
C99	10.6519930473#	0.0100038601#	53.3460008648#
H100	11.1636989451#	0.9592720384#	53.2872701434#
H101	9.5863320153#	0.1850130953#	53.3579369655#
H102	10.9249558605	-0.5915566631	52.4796236359
C103	11.0598723410	-0.6310999208	54.6899525080
H104	10.7433471116	-1.6818077174	54.6957546580
C105	10.3346468603	0.0681526540	55.8494601296

H106	9.2450911384	0.0065002139	55.7314318987
H107	10.6055817869	1.1329966521	55.8956060695
H108	10.5896294757	-0.3884684192	56.8114187889
C109	12.5781670768	-0.5726962796	54.9117014728
H110	13.1386130524	-1.0280116149	54.0883212518
H111	12.8652319841	-1.0940927109	55.8309587187
H112	12.9112633348	0.4718066220	54.9973794877
H113	13.4550771426#	-12.2303889725#	53.1883131065#
C114	13.9110028163#	-11.6210550813#	52.4219888766#
H115	14.8188489257	-11.1437228980	52.8008966288
H116	14.1085771285	-12.1961918422	51.5140845165
N117	12.8236123967	-10.6561136537	52.1939503244
H118	12.1716839630	-10.5267109589	52.9642250483
C119	12.6539467464	-9.8189577286	51.1717071100
N120	13.6563629803	-9.6431362306	50.2661125649
H121	13.4589563642	-8.8941094200	49.6089434129
H122	14.5636651267	-9.4882634691	50.7056255586
N123	11.4754786780	-9.2113342334	50.9852177916
H124	10.7946600065	-9.3189568135	51.7445187354
H125	11.5443799796	-8.1997740094	50.7349825167
H126	19.2055833693	-8.2173390044	61.7073587609
C127	19.5978928298#	-7.2298799107#	61.9900510831#
H128	20.6405927668	-7.3622346070	62.2974381163
C129	18.8329773360	-6.6901379481	63.1976555245
O130	19.3873512944	-6.2253071811	64.1910606570
C131	19.4918727491#	-6.2717981934#	60.7990648137#
H132	19.8515483447	-5.2785354372	61.0911235928
H133	18.4387530172	-6.1366441947	60.5325385751
C134	20.2505533020	-6.7577050037	59.5484794068
H135	20.0474598939	-7.8195019175	59.3651303253
H136	21.3361472082	-6.6886811414	59.7136118540
C137	19.9675675314	-6.0609350049	58.2091982650
O138	20.2029793742	-6.6152522340	57.1463887324
O139	19.4882647523	-4.8225647343	58.3256461182
N140	17.4797143692	-6.7539163853	63.0414626697
H141	17.1243588354	-7.0614161853	62.1442915931
C142	16.5368840816#	-6.0058840004#	63.8530429172#
H143	15.8590604950	-6.7030664941	64.3661533774
H144	17.1176560750	-5.4831047896	64.6173650469
C145	15.7086873257	-5.0171673487	62.9987941295
H146	16.3492022503	-4.1984607110	62.6532328895
H147	14.9340666156	-4.5791946356	63.6424393002
C148	15.0840814478	-5.7095059809	61.8036836168
C149	14.0001879319	-6.5893199489	61.9569508864
H150	13.5446234101	-6.7130143236	62.9382768106
C151	15.6426346127	-5.5471850722	60.5260363700
H152	16.4460317567	-4.8274960147	60.3872159164
C153	13.4888458355	-7.2905602167	60.8642233289
H154	12.6300913900	-7.9446766481	60.9885105792
C155	15.1445505673	-6.2633299325	59.4316985881

H156	15.5867753541	-6.1278897349	58.4486328883
C157	14.0672333823	-7.1377076123	59.5998840859
H158	13.6510510545	-7.6665819418	58.7485350002
H159	9.0037568144#	0.7050731996#	60.7752691711#
C160	8.1269795772#	0.1560810017#	60.4649990238#
H161	7.2836721157#	0.4592424005#	61.0677684635#
H269	18.2592772199#	-9.4987538239#	50.1505389989#
C270	17.5839764129#	-8.6559200934#	50.1520236221#
H271	17.2773632089#	-8.4419889044#	49.1387979570#
H273	23.2785566894	-4.2686859807	52.4551975095
H274	13.2748986226	-4.7533764318	58.1168571145
C275	6.5427374738	-8.5214460950	55.9526456524
H276	5.6202990340	-8.1064799948	55.5235125032
H277	7.2091325849	-8.7446095577	55.1137612009
H278	13.8856690297	-9.4944856780	56.7090481076

The BP86 optimized E_4 structure in **Figure 1.** # means fixed atom
Energies: E= -8167.676105, solv = -0.191471, disp = -230.83

Mo1	16.3745221555	-5.8284676841	53.4718468730
Fe2	9.8704124001	-7.1470098816	55.8808812325
Fe3	12.0168432620	-5.4241222244	55.8420848332
Fe4	11.3844243189	-6.5610214982	53.5702562028
Fe5	12.3791323344	-7.9993441572	55.4202384993
Fe6	14.7407474632	-7.5599433518	54.8387200577
Fe7	14.4461811119	-5.1133201430	55.2356795489
Fe8	13.7995137648	-6.0236247036	52.8568016032
C9	17.8252581532	-2.2352386855	54.1264462669
H10	18.0312331854	-1.2415812194	53.6984187265
H11	16.7305613581	-2.3075576497	54.2751726435
C12	18.2665954453	-3.3674337713	53.1588851855
C13	19.7331889694	-3.1494259643	52.7064958301
H14	20.3829165435	-3.2124508691	53.5957047392
H15	19.8099383108	-2.1273384801	52.2934944739
C16	20.1848035406	-4.1708739737	51.6388369111
H17	19.7025452884	-3.9774220948	50.6682951940
H18	19.8775611749	-5.1804336771	51.9753796833
C19	17.4415596396	-3.5692503991	51.8445275925
O20	17.0634586408	-4.8179882383	51.6498708254
O21	17.3218294649	-2.6410841148	51.0299907734
O22	18.1662325269	-4.6107954185	53.8892272931
C23	13.1071670055	-6.4465496784	54.6309261446
S24	11.5668671640	-7.1249595048	57.3518970224
S25	16.3019205189	-6.3160819707	55.7872679095
S26	10.0238435346	-5.0869073949	54.7573487195
S27	13.5369711881	-4.2954575691	57.1911941549
S28	14.0675850530	-9.7134474335	55.1561697367
S29	14.8275087421	-3.9931192042	53.3211614225
S30	10.5251943474	-8.6666540029	54.1808830535
S31	15.2380994111	-7.7004470957	52.5770331065

S32	12.0745158603	-6.0770363909	51.4158509910
H33	13.6351749360#	-2.1902102173#	49.0425570144#
C34	13.2979258889#	-2.0241168895#	50.0550232134#
H35	14.0189689948	-2.5424858019	50.7107628806
H36	13.3156042019	-0.9411273377	50.2796632485
N37	11.9543304097	-2.5883087445	50.2341551158
H38	11.3132819331	-2.5612532996	49.4439202394
C39	11.4193262305	-3.0362023242	51.3946266019
N40	12.0425972094	-2.8961138719	52.5630513845
H41	11.7526560739	-3.5230751803	53.3346451079
H42	13.0585246352	-2.7279451409	52.5829426361
N43	10.1195149173	-3.5050550600	51.3709337514
H44	10.0083186394	-4.2251844499	50.6472289924
H45	9.8737644247	-3.9470442502	52.2757380073
H46	7.9202575195	0.3966524082	59.4069694486
C47	8.2372840279	-1.3723799275	60.6816566441
H48	8.3975568038	-1.5522118284	61.7643503931
H49	7.2569948955	-1.8264582938	60.4367867485
C50	9.3039338978	-2.0936441984	59.9150814369
N51	10.6455244592	-1.9448396605	60.2428487101
C52	9.1577208379	-3.0092745702	58.8813349597
H53	8.2905004002	-3.4107679168	58.3598550050
C54	11.2936692109	-2.7613152196	59.4154873152
H55	12.3708834645	-2.9141511854	59.3780405592
N56	10.4398586768	-3.4252901574	58.5738696724
H57	19.2774661765	-4.3710746425	57.4335090345
H58	16.7107575277	-8.8813606829	50.7816249971
C59	18.3483868061	-7.4302321510	50.7005848595
H60	17.7094535181	-6.5321498689	50.6911156976
H61	19.2004450828	-7.2207271642	50.0236745669
C62	18.8914099224	-7.6133115072	52.0915587125
N63	18.2109284330	-7.2176187946	53.2456210467
C64	20.1067489074	-8.1607733751	52.4728702260
H65	20.9251432944	-8.5752030447	51.8877617988
C66	18.9900681481	-7.5141689215	54.2854925108
H67	18.7903740938	-7.2834549286	55.3295532615
N68	20.1431330357	-8.0979798230	53.8590198344
H69	20.9224508126	-8.3311088625	54.4705540985
H70	6.9488401142	-10.5717213650	56.9474681687
C71	6.1750532137#	-9.8249625395#	56.6900002953#
H72	5.5309556283#	-10.2322290215#	56.0424864027#
H73	5.6932327112#	-9.5371413521#	57.6127206361#
S74	8.0686459902	-7.7336821242	57.0677046982
H75	10.7087937698	-4.1157695564	57.8614188906
H76	18.4135352784	-4.3228057852	54.9049376361
C78	18.4637997838	-2.3121312760	55.5512588077
O79	18.7703552487	-3.5154954930	55.9794534155
O80	18.6001990822	-1.2597559909	56.2116786576
C81	21.6736016913	-4.2059782731	51.4311627975
O82	22.2939496647	-4.2108154887	50.3718703891

O83	22.3208176900#	-4.2658019842#	52.6398579665#
H84	15.7024659623#	-0.1996310679#	61.4912300362#
C85	14.8549931333#	-0.0829918662#	60.8320009256#
H86	13.9165020076#	-0.2867340019#	61.3260960181#
H87	14.8290646532	1.0013243937	60.5833925785
C88	15.0599868634#	-0.8751940580#	59.5441970379#
H89	14.3463795625	-0.5286156203	58.7737550321
H90	14.8314690420	-1.9422109486	59.7189409411
C91	16.4909210346	-0.7878632831	58.9924638785
H92	16.8910047678	0.2421955340	59.0984331773
H93	16.5160360334	-0.9999809511	57.9076130676
C94	17.4628882889	-1.7615566219	59.6872485653
O95	17.1852486605	-2.3635924098	60.7418228621
N96	18.6746105748	-1.8844138541	59.0562801658
H97	19.2247591469	-2.6937383383	59.3530947305
H98	18.7467171408	-1.6342066358	58.0480838698
C99	10.6519930238#	0.0100037989#	53.3460008750#
H100	11.1636989540#	0.9592720332#	53.2872701377#
H101	9.5863320154#	0.1850130962#	53.3579369739#
H102	10.9281404112	-0.5963432792	52.4703346588
C103	11.0483676589	-0.6607841876	54.6863658114
H104	10.7741068609	-1.7322512884	54.6430020489
C105	10.2620821401	-0.0363253477	55.8552066782
H106	9.1714308949	-0.1584128806	55.7148043159
H107	10.4736369380	1.0487951383	55.9410824766
H108	10.5294962809	-0.5098796058	56.8159189401
C109	12.5614227466	-0.5577781007	54.9503505644
H110	13.1591969265	-0.9464429498	54.1070930258
H111	12.8533320075	-1.1271511989	55.8500108231
H112	12.8547440174	0.5002708242	55.1053315508
H113	13.4550771306#	-12.2303889737#	53.1883130986#
C114	13.9110027931#	-11.6210550413#	52.4219888765#
H115	14.8699654607	-11.2132921504	52.7842849772
H116	14.0452178421	-12.1860684610	51.4844441895
N117	12.8852122720	-10.5852714505	52.2399208366
H118	12.3110662178	-10.3429142038	53.0649619076
C119	12.7493539187	-9.7552435840	51.1976021346
N120	13.7569721031	-9.6653135342	50.2657915634
H121	13.5778876836	-8.9239937683	49.5820212401
H122	14.6782309601	-9.5292728499	50.7044102059
N123	11.6022590682	-9.0718809833	51.0070933221
H124	10.9320908660	-9.1733186242	51.7908746265
H125	11.7547119948	-8.0200742272	50.8625378957
H126	19.1844583360	-8.2212743113	61.7143960240
C127	19.5978930863#	-7.2298798323#	61.9900510823#
H128	20.6518718344	-7.3794754125	62.2857756900
C129	18.8540303572	-6.6587310555	63.2042541829
O130	19.4174968194	-6.0973651292	64.1550780924
C131	19.4918727408#	-6.2717981580#	60.7990648772#
H132	19.8642163271	-5.2731762922	61.0911967130

H133	18.4283537863	-6.1272977551	60.5398449634
C134	20.2407384930	-6.7647429505	59.5404567753
H135	19.9966026548	-7.8247634623	59.3378382274
H136	21.3362924739	-6.7459428335	59.7148037177
C137	19.9980488348	-6.0355027867	58.2027935166
O138	20.2942754450	-6.5589773854	57.1260078983
O139	19.4721085431	-4.8067112830	58.3383387166
N140	17.4925614737	-6.7984995354	63.0937897373
H141	17.1295810665	-7.1792043689	62.2156625874
C142	16.5368840455#	-6.0058840614#	63.8530430146#
H143	15.9132094295	-6.6727265645	64.4833662698
H144	17.1210041204	-5.3555662544	64.5262297199
C145	15.6184173517	-5.1823308532	62.9072331750
H146	16.1900328205	-4.3480003467	62.4624880056
H147	14.8044572297	-4.7407494894	63.5136566186
C148	15.0557949075	-6.0635893705	61.8043904523
C149	14.0571771052	-7.0264121306	62.0704813112
H150	13.6033389320	-7.0706662317	63.0695422580
C151	15.6134170476	-6.0134300125	60.5068884275
H152	16.3515964841	-5.2351874149	60.2761844831
C153	13.6285360252	-7.9136603560	61.0705553375
H154	12.8305133670	-8.6340827067	61.2836236216
C155	15.1981391675	-6.9126744462	59.5070222303
H156	15.6364757793	-6.8577035298	58.5031939129
C157	14.2038473235	-7.8656450706	59.7888416539
H158	13.8478615246	-8.5335742477	58.9983982691
H159	9.0037568860#	0.7050731256#	60.7752690997#
C160	8.1269794130#	0.1560810308#	60.4649992665#
H161	7.2836720966#	0.4592425662#	61.0677683532#
H269	18.2592772010#	-9.4987538389#	50.1505389783#
C270	17.5839764984#	-8.6559201088#	50.1520234990#
H271	17.2773631776#	-8.4419889254#	49.1387979621#
H273	23.2780943207	-4.3015343469	52.4036989801
H274	13.9102792975	-5.2690423486	58.0775407554
C275	6.8419751633	-8.6161974944	55.9933112623
H276	6.0645602401	-7.8916281054	55.6809414149
H277	7.3654818431	-8.9544581577	55.0815772793
H278	14.1125780868	-9.8821198375	56.5100306390
H279	12.3867371216	-4.1027143428	55.1069830582
H280	14.3291353865	-7.6082005395	56.3064276357

The BP86 optimized structure for an approximate H-H TS
Energies: E= -8167.654396, solv = -0.191583, disp = -232.42

Mo1	16.4279874809	-5.8310201479	53.3851676171
Fe2	9.9377733592	-7.2676378086	55.7982138394
Fe3	12.3423246707	-6.0410073365	56.1311332102
Fe4	11.4594097128	-6.4992897934	53.6280045798
Fe5	12.4169033332	-8.3362096896	55.0692931140

Fe6	14.8572898947	-7.5884142355	54.8209542332
Fe7	14.5019372184	-5.1150499057	55.1159910650
Fe8	13.8490629229	-6.1031883047	52.7993643661
C9	17.7978847185	-2.2272818619	54.0569217401
H10	17.9745605309	-1.2231695244	53.6405546692
H11	16.7054221123	-2.3364440119	54.2014212207
C12	18.2785531805	-3.3357840139	53.0809916061
C13	19.7419227438	-3.0737526009	52.6430670482
H14	20.3853633545	-3.1286721720	53.5375849701
H15	19.7968056031	-2.0473155425	52.2379941970
C16	20.2273704159	-4.0768454200	51.5713438945
H17	19.7749990844	-3.8639798268	50.5908826784
H18	19.9128371881	-5.0916111171	51.8835917380
C19	17.4678866496	-3.5499599956	51.7591624964
O20	17.1024486800	-4.8021268300	51.5635768290
O21	17.3435084881	-2.6232572825	50.9435839003
O22	18.2077128229	-4.5872059755	53.7990112929
C23	13.2088967986	-6.6383049265	54.5617794781
S24	11.5745689232	-7.8649418936	57.1873643219
S25	16.4078660651	-6.2480723737	55.7117012215
S26	10.3754583558	-5.0651331459	55.0543323167
S27	13.3635003931	-4.0357793367	56.7992167602
S28	14.2944200496	-9.7683894560	55.2251618194
S29	14.8084862416	-4.0460806795	53.1875882921
S30	10.4417744129	-8.6189445031	53.8842826480
S31	15.3204149465	-7.7531859105	52.5499154650
S32	12.0699854313	-6.0685292414	51.4373925644
H33	13.6351749382#	-2.1902102198#	49.0425570154#
C34	13.2979258832#	-2.0241168885#	50.0550232166#
H35	14.0085193085	-2.5524140924	50.7140758275
H36	13.3242166854	-0.9423806257	50.2837065484
N37	11.9466226856	-2.5763050699	50.2263000433
H38	11.3048351919	-2.5143830910	49.4381529386
C39	11.3957385747	-3.0461578957	51.3701797032
N40	11.9891589284	-2.9416857953	52.5574205675
H41	11.6666366683	-3.6009204775	53.2909962865
H42	13.0074114016	-2.7856459754	52.6085926438
N43	10.0895590258	-3.5057283874	51.3067737013
H44	9.9954954640	-4.2210951491	50.5755953683
H45	9.8204803974	-3.9530421510	52.1974264128
H46	7.9176874477	0.4065298761	59.4094701338
C47	8.2500927610	-1.3745511845	60.6566141493
H48	8.4522195768	-1.5726878000	61.7292477221
H49	7.2653309097	-1.8333111181	60.4401340386
C50	9.2919552804	-2.0629956027	59.8268566344
N51	10.6448356330	-1.8355205447	60.0434473401
C52	9.1161491292	-2.9941293078	58.8117218179
H53	8.2334287790	-3.4444158573	58.3607428477
C54	11.2695447470	-2.6232236321	59.1709505382
H55	12.3472585415	-2.7120797862	59.0458159224

N56	10.3906054280	-3.3433065763	58.4043194771
H57	19.2683415882	-4.3878357691	57.4123518020
H58	16.7191924452	-8.8584070452	50.7979064545
C59	18.3797639289	-7.4294753390	50.6578105876
H60	17.7551818830	-6.5215683279	50.6386792943
H61	19.2196111465	-7.2512924695	49.9568682386
C62	18.9499875276	-7.5900098176	52.0385150001
N63	18.2783428005	-7.1932836290	53.1958111006
C64	20.1739794773	-8.1237421168	52.4098787915
H65	20.9892010537	-8.5362641408	51.8192955327
C66	19.0691757928	-7.4770663841	54.2307608484
H67	18.8750739990	-7.2442025722	55.2757568409
N68	20.2228073472	-8.0531436423	53.7955305688
H69	21.0084125793	-8.2814666792	54.4006375461
H70	6.9534258347	-10.5679028525	56.9432966621
C71	6.1750533052#	-9.8249624860#	56.6900002714#
H72	5.5309556213#	-10.2322290346#	56.0424864178#
H73	5.6932327070#	-9.5371413361#	57.6127206288#
S74	8.1171051656	-7.7740067528	57.0070090974
H75	10.6351692892	-4.0031255782	57.6552664911
H76	18.4367241108	-4.3029869342	54.8208104603
C78	18.4354232033	-2.3002112722	55.4829605590
O79	18.7574181753	-3.4982575460	55.9114662178
O80	18.5565569341	-1.2445709484	56.1429599897
C81	21.7225540548	-4.0974198023	51.4160985777
O82	22.3848777559	-4.0038341644	50.3872404022
O83	22.3208176458#	-4.2658019810#	52.6398579587#
H84	15.7024659566#	-0.1996310783#	61.4912300417#
C85	14.8549931579#	-0.0829918456#	60.8320009168#
H86	13.9165020077#	-0.2867340018#	61.3260960181#
H87	14.8266509551	1.0008216311	60.5813337294
C88	15.0599868400#	-0.8751940721#	59.5441970391#
H89	14.3222914958	-0.5541024965	58.7854023332
H90	14.8669376308	-1.9477016461	59.7330744214
C91	16.4784694257	-0.7434703728	58.9638971971
H92	16.8388651197	0.3027848942	59.0532368437
H93	16.4921905210	-0.9651058353	57.8807300940
C94	17.4998736029	-1.6680644977	59.6548356076
O95	17.2803141849	-2.2229774550	60.7495827773
N96	18.6820776008	-1.8050488504	58.9768676058
H97	19.2676443652	-2.5798750236	59.2960858745
H98	18.7224632048	-1.5876782362	57.9579628252
C99	10.6519930337#	0.0100037918#	53.3460008434#
H100	11.1636989568#	0.9592720320#	53.2872701435#
H101	9.5863320165#	0.1850131009#	53.3579369917#
H102	10.9275507152	-0.5957898885	52.4693516321
C103	11.0635067757	-0.6545780725	54.6828050199
H104	10.7782196954	-1.7241064723	54.6561370145
C105	10.3089276879	-0.0065180034	55.8589802743
H106	9.2146722937	-0.1244266096	55.7479039854

H107	10.5282768433	1.0786729405	55.9186774834
H108	10.5983939983	-0.4628649368	56.8216634677
C109	12.5825325820	-0.5608201138	54.9093768115
H110	13.1563882224	-0.9602310494	54.0541611599
H111	12.8936261640	-1.1266897317	55.8043649143
H112	12.8866046944	0.4963796819	55.0464756867
H113	13.4550771382#	-12.2303889833#	53.1883130956#
C114	13.9110027901#	-11.6210550487#	52.4219888901#
H115	14.8526339213	-11.1895584849	52.8031454419
H116	14.0878094704	-12.2060382490	51.5034881351
N117	12.8826475701	-10.6040036084	52.1739821765
H118	12.2777513933	-10.3357377386	52.9705122512
C119	12.8174357003	-9.7760922743	51.1218880983
N120	13.8652034184	-9.7267864900	50.2327895546
H121	13.7391643664	-8.9906018877	49.5326947169
H122	14.7751939265	-9.6246948833	50.7012604045
N123	11.7009791177	-9.0602213055	50.8761081516
H124	10.9888051825	-9.1512974869	51.6252283535
H125	11.8782651375	-8.0099312311	50.7644116471
H126	19.2099954434	-8.2283705442	61.7012844866
C127	19.5978931795#	-7.2298797483#	61.9900511234#
H128	20.6497516264	-7.3627418734	62.3001886616
C129	18.8296690857	-6.7078457826	63.2072541644
O130	19.3756303881	-6.2966965018	64.2422660395
C131	19.4918726513#	-6.2717982168#	60.7990648409#
H132	19.8543531929	-5.2696853051	61.0930461135
H133	18.4300075093	-6.1294427155	60.5283700032
C134	20.2488524237	-6.7542103457	59.5407821380
H135	20.0128117815	-7.8155435364	59.3350079990
H136	21.3437312986	-6.7293205333	59.7180171728
C137	20.0093673594	-6.0283122884	58.1992911070
O138	20.3354150604	-6.5451853016	57.1288322904
O139	19.4523815579	-4.8100356502	58.3217285233
N140	17.4723425431	-6.7304858085	63.0143192068
H141	17.1212673265	-6.9838578482	62.0872969099
C142	16.5368840311#	-6.0058841351#	63.8530430003#
H143	15.8454379337	-6.7192201531	64.3472645335
H144	17.1328713534	-5.5159857807	64.6421312003
C145	15.7073377977	-4.9788505651	63.0357671284
H146	16.3533654200	-4.1449037723	62.7081145096
H147	14.9290720842	-4.5578136467	63.7016498117
C148	15.0872846597	-5.6551830736	61.8270350964
C149	14.0327613982	-6.5821075059	61.9776764572
H150	13.5730862140	-6.7210240055	62.9657422544
C151	15.6560040922	-5.4735376041	60.5464096053
H152	16.4400976568	-4.7166179790	60.4152897921
C153	13.5691316311	-7.3245127446	60.8812240589
H154	12.7369443220	-8.0274079503	61.0021012502
C155	15.2032536490	-6.2303026186	59.4493263260
H156	15.6643830671	-6.0983997912	58.4623005119

C157	14.1661733611	-7.1637929682	59.6192465795
H158	13.7955393354	-7.7380186692	58.7660245658
H159	9.0037568924#	0.7050731213#	60.7752690891#
C160	8.1269793736#	0.1560810443#	60.4649992459#
H161	7.2836721133#	0.4592425665#	61.0677683765#
H269	18.2592772214#	-9.4987538225#	50.1505389768#
C270	17.5839764221#	-8.6559201349#	50.1520235453#
H271	17.2773632142#	-8.4419889106#	49.1387979540#
H273	23.2873279585	-4.2732836758	52.4425296980
H274	14.1899759027	-4.4706821626	57.7963728839
C275	6.8300828290	-8.6175424295	55.9782204860
H276	6.0503810855	-7.8787045787	55.7068202114
H277	7.3070864173	-8.9545588191	55.0411245777
H278	14.1934533272	-9.8546396143	56.5839690443
H279	13.6882710199	-6.7194808650	56.7802918449
H280	14.4356142948	-7.4207512839	56.3807421004

The BP86 optimized structure for E_4-H_2

Energies: E= -8167.537537, solv = -0.192262, disp = -226.92

Mo1	16.2483435426	-5.7540525960	53.3489244179
Fe2	9.7627163010	-7.1184137254	55.7063492721
Fe3	11.9327266329	-5.5543341480	55.6898309384
Fe4	11.2832059306	-6.5239657862	53.3828931610
Fe5	12.2630326928	-8.0265487859	55.2048403942
Fe6	14.5839544731	-7.5918870693	54.3498904177
Fe7	14.3320891624	-5.1705492855	55.0879314175
Fe8	13.6634471576	-5.8551717237	52.6434960871
C9	17.7788982267	-2.1795118119	54.1093635668
H10	18.0192055286	-1.1971910175	53.6738363505
H11	16.6798507690	-2.2235567853	54.2372885555
C12	18.2078443448	-3.3322660914	53.1643357278
C13	19.7008741615	-3.1792147326	52.7666534275
H14	20.3165144225	-3.2835360122	53.6763725603
H15	19.8368934029	-2.1548032615	52.3748573001
C16	20.1473017268	-4.1995954099	51.6967123993
H17	19.6500090667	-4.0111211672	50.7328581677
H18	19.8539898923	-5.2123911035	52.0344455994
C19	17.4167456726	-3.4835420604	51.8253832355
O20	17.0028800666	-4.7135224258	51.5896453438
O21	17.3424328650	-2.5325044776	51.0325601210
O22	18.0192869360	-4.5667325722	53.8955375527
C23	13.0099559147	-6.4590083137	54.3873287371
S24	11.4629600044	-7.2263624553	57.1899290793
S25	16.0819692003	-6.4698386261	55.6384533047
S26	9.9438807135	-4.9937267657	54.5990585976
S27	13.2698307173	-3.8943602199	56.6740826960
S28	14.0052223523	-9.6036342210	55.3551751639
S29	14.7029209970	-3.8955339653	53.2896145986

S30	10.4023628550	-8.6428529472	53.9780123388
S31	15.1389478817	-7.4753555576	52.1506361013
S32	11.9224248703	-6.0198795221	51.2128135126
H33	13.6351750606#	-2.1902102712#	49.0425570648#
C34	13.2979256768#	-2.0241168384#	50.0550231609#
H35	13.9583424267	-2.6106388925	50.7166293805
H36	13.3854628177	-0.9512939983	50.3072068992
N37	11.9088035260	-2.4932658824	50.1774883016
H38	11.3126699838	-2.4386791863	49.3540067777
C39	11.3008712695	-2.9746230486	51.2855003484
N40	11.8476327158	-2.9001275455	52.4965342480
H41	11.4810024808	-3.5596111737	53.2099720377
H42	12.8611628771	-2.7382478835	52.5949995348
N43	9.9944797123	-3.4184555101	51.1584947409
H44	9.9407061552	-4.1476194652	50.4356352677
H45	9.6758006329	-3.8541813344	52.0412015769
H46	7.9078592352	0.4084767034	59.4122687149
C47	8.2531865921	-1.3776147322	60.6720962142
H48	8.7632123682	-1.5798963578	61.6315808337
H49	7.2244293670	-1.7786245532	60.7910453020
C50	8.9636893235	-2.1964979741	59.6258624008
N51	9.9714254066	-3.0761875375	60.0009989545
C52	8.6825213946	-2.3485009748	58.2719696228
H53	7.9624799257	-1.8750388894	57.6065782237
C54	10.2884482364	-3.7431260786	58.8930267566
H55	11.0364307378	-4.5292842527	58.8012632866
N56	9.5402891331	-3.3381851417	57.8216910893
H57	19.1946136520	-4.3471541710	57.4615636018
H58	16.7082717180	-8.8835162513	50.7755660224
C59	18.3289862364	-7.4151922459	50.6911701111
H60	17.6815420570	-6.5234832766	50.6452284018
H61	19.1966415941	-7.2137980808	50.0323218829
C62	18.8269384021	-7.5659281143	52.0995161571
N63	18.0890546833	-7.1689341743	53.2158037875
C64	20.0359065376	-8.0850832887	52.5366412373
H65	20.8847464223	-8.4925050017	51.9914620481
C66	18.8318337764	-7.4372588970	54.2906280396
H67	18.5822845756	-7.2005206810	55.3219514107
N68	20.0122284677	-8.0028100659	53.9223116217
H69	20.7701625609	-8.2004379745	54.5725573456
H70	6.9720339396	-10.5496817534	56.9347174644
C71	6.1750535847#	-9.8249626408#	56.6900005170#
H72	5.5309555837#	-10.2322287892#	56.0424863010#
H73	5.6932326724#	-9.5371413351#	57.6127206104#
S74	7.9945106259	-7.6437364544	56.9534980604
H75	9.6067763193	-3.7320442110	56.8728218801
H76	18.2376732689	-4.2866715813	54.9178406381
C78	18.3960041053	-2.2643406519	55.5398662897
O79	18.6218966891	-3.4731123052	55.9978590762
O80	18.5932573370	-1.2037487337	56.1741372155

C81	21.6326183906	-4.2213996734	51.4532364404
O82	22.2199925077	-4.2280680615	50.3753289616
O83	22.3208176274#	-4.2658019779#	52.6398579821#
H84	15.7024659542#	-0.1996310824#	61.4912300439#
C85	14.8549931724#	-0.0829918355#	60.8320009132#
H86	13.9165020076#	-0.2867340019#	61.3260960181#
H87	14.8237270106	1.0009930069	60.5804166556
C88	15.0599868076#	-0.8751940751#	59.5441970446#
H89	14.3083624427	-0.5760215242	58.7902244537
H90	14.8959692499	-1.9519104984	59.7331023112
C91	16.4695325817	-0.6952691990	58.9519006162
H92	16.7642938371	0.3750611646	58.9776365963
H93	16.4927786903	-0.9790806421	57.8837980810
C94	17.5519736291	-1.5082378370	59.6895379072
O95	17.3851858826	-1.9916373210	60.8275754846
N96	18.7293606699	-1.6312581942	59.0021562521
H97	19.3705406732	-2.3323074146	59.3780799539
H98	18.7551123141	-1.4772584534	57.9702419918
C99	10.6519930294#	0.0100038407#	53.3460008574#
H100	11.1636989617#	0.9592720302#	53.2872701557#
H101	9.5863320166#	0.1850131036#	53.3579369620#
H102	10.9271748845	-0.5974640731	52.4720697638
C103	11.0735510301	-0.6381064015	54.6863327428
H104	10.7806397432	-1.7059927543	54.6812464921
C105	10.3399928982	0.0380746113	55.8596464947
H106	9.2420233182	-0.0387895880	55.7450644424
H107	10.5972846176	1.1152011547	55.9172782130
H108	10.6067702509	-0.4286460383	56.8235804519
C109	12.5957431889	-0.5481027840	54.8937601821
H110	13.1596827097	-0.9877843107	54.0518119667
H111	12.9109595587	-1.0782452745	55.8088335205
H112	12.9089988123	0.5118259779	54.9834247420
H113	13.4550771461#	-12.2303889840#	53.1883130995#
C114	13.9110028044#	-11.6210550657#	52.4219888785#
H115	14.8510225905	-11.1841364030	52.8039878020
H116	14.0888874131	-12.2038934457	51.5032767800
N117	12.8804690765	-10.6016904391	52.1808118164
H118	12.2727572936	-10.3408299533	52.9746995677
C119	12.7881007712	-9.7865692772	51.1180988166
N120	13.8071409650	-9.7609762006	50.1944529115
H121	13.6640860149	-9.0307500829	49.4908129581
H122	14.7321712828	-9.6521132620	50.6292071250
N123	11.6683739262	-9.0723841393	50.8948180812
H124	10.9748450599	-9.1375638006	51.6635438472
H125	11.8240268491	-8.0270258155	50.7114614306
H126	19.2027816100	-8.2264635561	61.7041837753
C127	19.5978928335#	-7.2298798911#	61.9900510898#
H128	20.6498082343	-7.3691681762	62.2972144067
C129	18.8342480805	-6.7031596150	63.2088092458
O130	19.3817134817	-6.2873983691	64.2409729996

C131	19.4918727293#	-6.2717982178#	60.7990648061#
H132	19.8809329373	-5.2778222673	61.0871402139
H133	18.4282784764	-6.1046428167	60.5497306066
C134	20.2090347117	-6.7598952370	59.5211317426
H135	19.9459311815	-7.8139024510	59.3099793734
H136	21.3086178755	-6.7583440849	59.6680643717
C137	19.9440020174	-6.0091398646	58.1974482685
O138	20.2388544569	-6.5103679059	57.1099371158
O139	19.3988540304	-4.7908759378	58.3573741062
N140	17.4757604407	-6.7333952667	63.0203333179
H141	17.1288037528	-6.9763167254	62.0893072836
C142	16.5368840868#	-6.0058840053#	63.8530429226#
H143	15.8127373672	-6.7170213210	64.3010188810
H144	17.1210697223	-5.5595043108	64.6755934147
C145	15.7623251356	-4.9250828988	63.0474076207
H146	16.4248733805	-4.0650242971	62.8444234641
H147	14.9287087727	-4.5591807273	63.6780675733
C148	15.2466077684	-5.4773855463	61.7280314378
C149	14.1975409670	-6.4232202804	61.6797214063
H150	13.6739905076	-6.6996904022	62.6049564505
C151	15.8890015019	-5.1161019959	60.5231639865
H152	16.6687267617	-4.3442568179	60.5439444693
C153	13.8053629235	-6.9991903874	60.4599859274
H154	12.9707196825	-7.7083945875	60.4267193144
C155	15.5084205971	-5.7041626421	59.3032917236
H156	16.0140046307	-5.4200081798	58.3735882318
C157	14.4701964450	-6.6515614604	59.2693745063
H158	14.1585033986	-7.0932374115	58.3163466169
H159	9.0037568171#	0.7050731809#	60.7752691965#
C160	8.1269795689#	0.1560810125#	60.4649989252#
H161	7.2836721272#	0.4592423717#	61.0677684941#
H269	18.2592772350#	-9.4987538118#	50.1505389908#
C270	17.5839763606#	-8.6559201050#	50.1520236561#
H271	17.2773632328#	-8.4419888992#	49.1387979509#
H273	23.2693158551	-4.2900575032	52.3694546854
H274	13.5908786972	-4.6226998560	57.7853416567
C275	6.7751334207	-8.6048469795	55.9525520256
H276	5.9552872356	-7.9230560164	55.6505326839
H277	7.2776148360	-8.9442147318	55.0294541156
H278	14.4521678257	-9.3185291943	56.6127560003

The B3LYP optimized structure for A_4-N_2 in Figure 4
Energies: E= -8028.671524, solv = -0.195915, disp = -235.96

Mo1	16.4828623181	-5.6823684169	53.2479254599
Fe2	9.1179852696	-6.7758148947	55.4208663320
Fe3	11.4603600748	-5.3632758831	55.3585087217
Fe4	10.8991241872	-6.5413404504	52.8319160864
Fe5	11.8666205900	-8.0536170890	54.9748476120

Fe6	14.3704248213	-7.5948225012	54.1935112748
Fe7	14.1440468036	-5.1642072681	54.9429343425
Fe8	13.3585411796	-5.7826779847	52.2236210667
C9	17.8123626574	-2.0788574801	54.2302334611
H10	17.9970754188	-1.0761595265	53.8405432392
H11	16.7270839015	-2.1891198502	54.3503688584
C12	18.3053679684	-3.1568954544	53.2410226062
C13	19.7856910607	-2.9180360744	52.8616823319
H14	20.4011069521	-3.1290445267	53.7409298966
H15	19.9040529744	-1.8588257656	52.6083363701
C16	20.2489940758	-3.7733653367	51.6620678026
H17	19.8083509123	-3.4098588286	50.7327298081
H18	19.9251087838	-4.8086205204	51.8186021326
C19	17.5323951019	-3.2807507269	51.8957965122
O20	17.2099850419	-4.5105549225	51.5896920582
O21	17.3983190503	-2.2955986246	51.1749067319
O22	18.1708184422	-4.4285411064	53.9066098555
C23	12.6865646708	-6.4455021773	54.1289126533
S24	13.0062326469	-4.1116665078	56.7816036855
S25	16.0153618757	-6.4862516041	55.5207669423
S26	9.5724019718	-4.8225018899	54.0553749230
S27	13.5846715050	-9.5557313721	55.0672180292
S28	14.6884688529	-3.8965581893	53.1391374665
S29	9.9172890111	-8.5274441842	53.7984200878
S30	15.1583266955	-7.3324484675	52.0037150170
S31	11.6438974584	-6.0778037034	50.6677795771
H32	13.6351744979#	-2.1902108077#	49.0425561294#
C33	13.2979238585#	-2.0241171884#	50.0550234240#
H34	13.8947793154	-2.6597513943	50.7139398370
H35	13.4359258264	-0.9729291241	50.3312259557
N36	11.8834539903	-2.4068633800	50.0874465283
H37	11.3661560622	-2.3683548277	49.2216144703
C38	11.2005448850	-2.8843141185	51.1330621542
N39	11.7001696781	-2.8873255884	52.3569863166
H40	11.2019720041	-3.4403134662	53.0621815545
H41	12.6970213267	-2.7548628377	52.5269124085
N42	9.9021530928	-3.2741682805	50.9232181689
H43	9.8477959142	-3.9513290049	50.1637672384
H44	9.4935594797	-3.7020786258	51.7589767942
H45	7.9323733854	0.3890381182	59.4126098269
C46	8.3365478346	-1.3548130834	60.6920150406
H47	8.1966254474	-1.5716313260	61.7579542880
H48	7.5613894678	-1.9112143507	60.1521349787
C49	9.6993199017	-1.8484273140	60.3187508756
N50	10.7053094746	-1.9157651491	61.2656430520
C51	10.1797661195	-2.2470177566	59.0922596275
H52	9.7141113985	-2.3600233749	58.1256672549
C53	11.7670790298	-2.3536144296	60.6156512523
H54	12.7404530391	-2.5461638069	61.0453006993
N55	11.5039708056	-2.5606868409	59.2979202628
H56	19.2322167186	-4.3078032649	57.5145411537

H57	16.7263501410	-8.8550584274	50.7888243741
C58	18.3807511512	-7.4221635437	50.6305515280
H59	17.7633154522	-6.5227000629	50.5729102742
H60	19.2207457168	-7.2711447175	49.9381433009
C61	18.9300079347	-7.5413503662	52.0189704535
N62	18.2352348062	-7.1019819665	53.1372118633
C63	20.1270815095	-8.0690939422	52.4382324472
H64	20.9452724474	-8.5100686499	51.8900695506
C65	18.9876543589	-7.3567430181	54.1964920794
H66	18.7611251729	-7.1016234116	55.2199931533
N67	20.1383582607	-7.9522167774	53.8177953652
H68	20.8928758336	-8.1745218141	54.4502376223
H69	6.9382483265	-10.5716730055	56.9406189088
C70	6.1750554245#	-9.8249629312#	56.6900012825#
H71	5.5309554731#	-10.2322278877#	56.0424858443#
H72	5.6932323134#	-9.5371413313#	57.6127204220#
S73	8.1018222096	-7.9083834655	57.1611659263
H74	12.1363810291	-2.9169896503	58.5858084035
H75	18.3623617281	-4.2184122745	54.9096316084
C76	18.4348431944	-2.1898255230	55.6519989917
O77	18.6961101322	-3.3879274563	56.0727583839
O78	18.6003052719	-1.1448514313	56.3038265462
C79	21.7391881196	-3.7813010466	51.5111302661
O80	22.4021145175	-3.4323753946	50.5543666487
O81	22.3208168657#	-4.2658019472#	52.6398595893#
H82	15.7024655402#	-0.1996319671#	61.4912314205#
C83	14.8549939627#	-0.0829907286#	60.8320019266#
H84	13.9165015893#	-0.2867338795#	61.3260937710#
H85	14.8235908583	0.9908962859	60.5818225187
C86	15.0599852763#	-0.8751946093#	59.5441989644#
H87	14.3369976753	-0.5481571687	58.7863786115
H88	14.8660007570	-1.9395961722	59.7221962656
C89	16.4807265297	-0.7388567001	58.9832007796
H90	16.8136865097	0.3090642851	59.0266081780
H91	16.5185152695	-1.0092627962	57.9218340818
C92	17.5098639459	-1.6000048614	59.7283398999
O93	17.2646164027	-2.1509083890	60.8078974324
N94	18.7199888519	-1.6905784817	59.1154825893
H95	19.3207648276	-2.4259009535	59.4688428035
H96	18.7942410290	-1.4782847167	58.1094913634
C97	10.6519928840#	0.0100026994#	53.3460012113#
H98	11.1636988107#	0.9592721561#	53.2872698723#
H99	9.5863320810#	0.1850135266#	53.3579374751#
H100	10.9225763210	-0.5883793139	52.4774646866
C101	11.0617907341	-0.6407932349	54.6836636613
H102	10.7321514992	-1.6880535208	54.6852877954
C103	10.3521880850	0.0666022989	55.8487555318
H104	9.2615975862	-0.0149044681	55.7570374676
H105	10.6079674293	1.1357128640	55.8719622766
H106	10.6393842697	-0.3634451879	56.8146118880
C107	12.5828225232	-0.6036340069	54.8879458445

H108	13.1282364034	-1.0752407046	54.0634901086
H109	12.8750130541	-1.1224295152	55.8075992288
H110	12.9345716226	0.4352553703	54.9627585357
H111	13.4550782458#	-12.2303881420#	53.1883144238#
C112	13.9110011147#	-11.6210559043#	52.4219862303#
H113	14.7842347215	-11.1032194230	52.8279319152
H114	14.1638285885	-12.2153157867	51.5396819151
N115	12.8097262762	-10.7017852493	52.1110082812
H116	12.2667519362	-10.4259779248	52.9308668305
C117	12.6879970385	-9.8927492191	51.0617343094
N118	13.7084059601	-9.7683290639	50.1662425256
H119	13.5417910760	-9.0459582877	49.4724044990
H120	14.6148717915	-9.6317688716	50.6091471337
N121	11.5248505549	-9.2688732961	50.8283029999
H122	10.8148077057	-9.3635162964	51.5582998159
H123	11.5832341565	-8.2813003964	50.5037055585
H124	19.1979626076	-8.2159703844	61.7130496938
C125	19.5978940310#	-7.2298787586#	61.9900508988#
H126	20.6404514083	-7.3680800013	62.2951219857
C127	18.8379094031	-6.6785704344	63.1953676320
O128	19.3924791249	-6.1980406397	64.1806047207
C129	19.4918709091#	-6.2717989832#	60.7990629473#
H130	19.8796612002	-5.2880273113	61.0880738027
H131	18.4362449473	-6.1085822364	60.5582535016
C132	20.2051266299	-6.7533211164	59.5224700085
H133	19.9503144023	-7.7981620194	59.3076478375
H134	21.2960068648	-6.7384961422	59.6637653361
C135	19.9245681884	-5.9938656538	58.2150458938
O136	20.1636104060	-6.4981555650	57.1283787179
O137	19.4398539649	-4.7646844629	58.3870997771
N138	17.4835209998	-6.7509095959	63.0448846898
H139	17.1286726863	-7.0741675208	62.1538704810
C140	16.5368850183#	-6.0058848485#	63.8530403554#
H141	15.8365564364	-6.7061216628	64.3300743412
H142	17.1085115949	-5.5176708090	64.6461191745
C143	15.7438790858	-4.9703596766	63.0155241673
H144	16.3859957316	-4.1137612398	62.7849686902
H145	14.9147528307	-4.5997403157	63.6337956271
C146	15.2302577877	-5.5650615685	61.7167084533
C147	14.2508433876	-6.5717271285	61.7063792852
H148	13.7788451590	-6.8746057877	62.6396132022
C149	15.8099285890	-5.1794935995	60.4972538720
H150	16.5434341162	-4.3772506251	60.4902755996
C151	13.8784451069	-7.1945274824	60.5123902978
H152	13.1201599428	-7.9723696879	60.5209388182
C153	15.4449183300	-5.8069535542	59.3020401140
H154	15.9103217911	-5.5137677958	58.3652960858
C155	14.4860767140	-6.8235947493	59.3078300173
H156	14.2308238895	-7.3275446530	58.3798044844
H157	9.0037560058#	0.7050734217#	60.7752692958#
C158	8.1269804859#	0.1560798882#	60.4649992489#

H159	7.2836720913#	0.4592419839#	61.0677701412#
H161	18.2592787252#	-9.4987524176#	50.1505399206#
C162	17.5839748013#	-8.6559204990#	50.1520276233#
H163	17.2773626378#	-8.4419891117#	49.1387987840#
H164	23.2817257051	-4.2307479730	52.4763442051
H167	13.1840102905	-5.1320865560	57.6512083419
C168	6.8284382047	-8.6229164446	56.0383438886
H169	7.3021630651	-8.9234282743	55.0972031921
H170	6.0711984477	-7.8637025610	55.8038573911
H173	7.1493939899#	-5.8354098776#	59.8218943989#
C174	6.9590151485#	-4.8009334498#	59.5000035385#
H175	5.9039259982#	-4.4903549997#	59.5181448765#
H176	7.4947683113	-4.1641468785	60.2178086426
C177	7.4747921753	-4.5037460688	58.0898121002
H178	8.5235807514	-4.8125866577	57.9828261359
H179	7.4332337301	-3.4219313316	57.9114494603
O180	6.6702747049	-5.1003672971	57.0807316794
H181	6.9622496053	-6.0384050563	57.0540748593
H184	10.4127182259	-6.1573818490	56.4971858857
N185	11.6903149939	-8.3212801274	57.2578782231
N186	11.5651436640	-8.6445544923	58.3075191383
